# IL-16 Promotes *T. whipplei* Replication by Inhibiting Phagosome Conversion and Modulating Macrophage Activation

**DOI:** 10.1371/journal.pone.0013561

**Published:** 2010-10-21

**Authors:** Eric Ghigo, Abdoulaye Oury Barry, Lionel Pretat, Khatoun Al Moussawi, Benoît Desnues, Christian Capo, Hardy Kornfeld, Jean-Louis Mege

**Affiliations:** 1 URMITE, CNRS UMR 6236-IRD 3R198, Université de la Méditerranée, Marseille, France; 2 Division of Pulmonary and Critical Care Medicine, Department of Medicine, University of Massachusetts Medical School, Worcester, Massachusetts, United States of America; BMSI-A*STAR, Singapore

## Abstract

The replication of *Tropheryma whipplei* (the agent of Whipple's disease) within human macrophages is associated with the expression of IL-16, a cytokine known for its chemotactic and inflammatory properties. In this study, we asked whether IL-16 acts on *T. whipplei* replication by interfering with the endocytic pathway. We observed that in macrophages, *T. whipplei* was located within late phagosomes that were unable to fuse with lysosomes; in monocytes, *T. whipplei* was eliminated in phagolysosomes. Moreover, adding IL-16 to monocytes induced bacterial replication and inhibited phagolysosome formation. On the other hand, blocking IL-16 activity, either with anti-IL-16 antibodies in human macrophages or by using murine IL-16^−/−^ bone marrow-derived macrophages, inhibited *T. whipplei* replication and rescued phagolysosome biogenesis. Furthermore, we propose that IL-16-mediated interference with the endocytic pathway is likely related to macrophage activation. First, IFNγ induced *T. whipplei* elimination and phagolysosome formation and inhibited IL-16 production by macrophages. Second, the full transcriptional response of murine macrophages to *T. whipplei* showed that *T. whipplei* specifically modulated the expression of 231 probes in IL-16^−/−^ macrophages. Gene Ontology analysis revealed that 10 of 13 over-represented terms were linked to immune responses, including proinflammatory transcriptional factors of the NF-κB family. Our results demonstrated a previously unreported function for IL-16 in promoting bacterial replication through inhibited phagolysosome biogenesis and modulated macrophage activation program.

## Introduction

Whipple's disease (WD) is a chronic multisystemic infection caused by *Tropheryma whipplei* [1]. The classical manifestations of WD are weight loss, diarrhea, polyarthralgia, fever, and lymphadenopathy. In addition, cardiac and central nervous system symptoms may also be associated with WD [2]. The course of WD is fatal unless antibiotic treatment is initiated [3]. Specific immune deficiencies and genetic traits have been suggested to be involved in WD development. Indeed, impaired interferon (IFN) γ production associated with interleukin (IL)-12 deficiency and Th2 repolarization of the immune response has been considered to be critical for WD physiopathology [2], [4]. Consistent with these observations, we previously found that macrophages from intestinal lesions are polarized into alternatively activated macrophages, also known as M2 macrophages [5]. Furthermore, we have shown that *T. whipplei* replication in human macrophages is related to IL-16 production [6]. IL-16, constitutively produced as cytosolic pro-IL-16, is secreted after caspase-3-mediated processing [7], [8], [9] by numerous cell types [10], [11], [12]. IL-16 is a chemoattractant for CD4-expressing immune cells, such as T cells [10], monocytes [13], dendritic cells [14] and eosinophils [15]. Aside from its role as a chemoattractant, IL-16 may also be involved in the innate immune response because it favors the production of inflammatory cytokines by monocytes [16] and may act on antigen-presenting cells as well [17]. IL-16 is also known to modulate the adaptive immune response by favoring Th1 responses. Indeed, IL-16 primes T cells to IL-15 production and CD25 expression, and thus renders them more susceptible to the presence of IL-2 [18].

One of the microbicidal mechanisms of macrophages is based on the formation of phagolysosomes. Specifically, macrophages internalize microorganisms into phagosomes, which undergo extensive remodeling involving an active exchange of material with plasma membrane, endosomes, lysosomes, Golgi- and endoplasmic-derived vesicles. Phagosomes fuse with early endosomes and then with late endosomes, as demonstrated by the acquisition of specific markers such as early endosome antigen-1, the small GTPases Rab5 and Rab7, and lysosome-associated membrane protein (Lamp)-1, respectively. Finally, late phagosomes fuse with lysosomes and acquire hydrolase enzymes such as cathepsin D [19]. Microorganisms are destroyed in these phagolysosomes, which are associated with both Lamp-1 and cathepsin D [20]. However, several types of pathogenic microorganisms have been shown to manipulate host cell organelles and membrane trafficking processes to survive and replicate within host cells [21], [22]. For example, *Salmonella* resides in an atypical phagosome that is neither an early nor a late phagosome [23]. *Mycobacterium*-containing phagosomes fuse with early endosomes but are unable to fuse with late endosomes [24], [25]. *Coxiella burnetii*, the agent of Q fever, survives in macrophages within an acidic late phagosome that does not fuse with lysosomes [26].

It is well-established that the microbicidal activity of monocytes/macrophages is regulated by cytokines that polarize macrophages [27]. IFNγ, a cytokine known to confer microbicidal competence to macrophages, controls *Listeria monocytogenes* infection and its clearance [28]. However, only a few reports have attempted to address the relationship between cytokines and phagosome conversion. It has been shown that treatment of macrophages with IFNγ allows the elimination of mycobacteria through the conversion of bacterial phagosomes to phagolysosomes [29], [30]. IFNγ triggers the listericidal competence of macrophages by up-regulating the small GTPase activity required for phagosome conversion [31]. IFNγ stimulates *C. burnetii* elimination and induces the maturation of *C. burnetii* phagosomes in phagolysosomes [26]. Conversely, IL-10, an immunosuppressive cytokine, promotes the intracellular localization of mycobacteria within phagosomes that are unable to fuse with lysosomes [29] and stimulates *C. burnetii* replication by increasing the ability of *C. burnetii* to traffic into an acidified late phagosome [32], [33].

We have previously shown that *T. whipplei* is localized within an acidic compartment associated with Lamp-1 unable to fuse with lysosomes [34] and that *T. whipplei* replication in human macrophages is related to IL-16 production [6]. However, the mechanisms triggered by IL-16 that affect the intracellular fate of *T. whipplei* remain largely understudied. In this study, we found that IL-16 was required for *T. whipplei* replication within late phagosomes in both human and murine macrophages. The lack of IL-16 led to *T. whipplei* elimination within phagolysosomes. The effect of IL-16 on *T. whipplei* replication in late phagosomes was repressed by inhibiting IL-16 production by IFNγ. Microarray analysis of IL-16^−/−^ macrophages indicated roles for IL-16 in the innate immune response and the NF-κB pathway. Taken together, our study demonstrates novel functions of IL-16 in intracellular trafficking and macrophage regulation.

## Results

### Intracellular localization of *T. whipplei*

**Figure 1:**
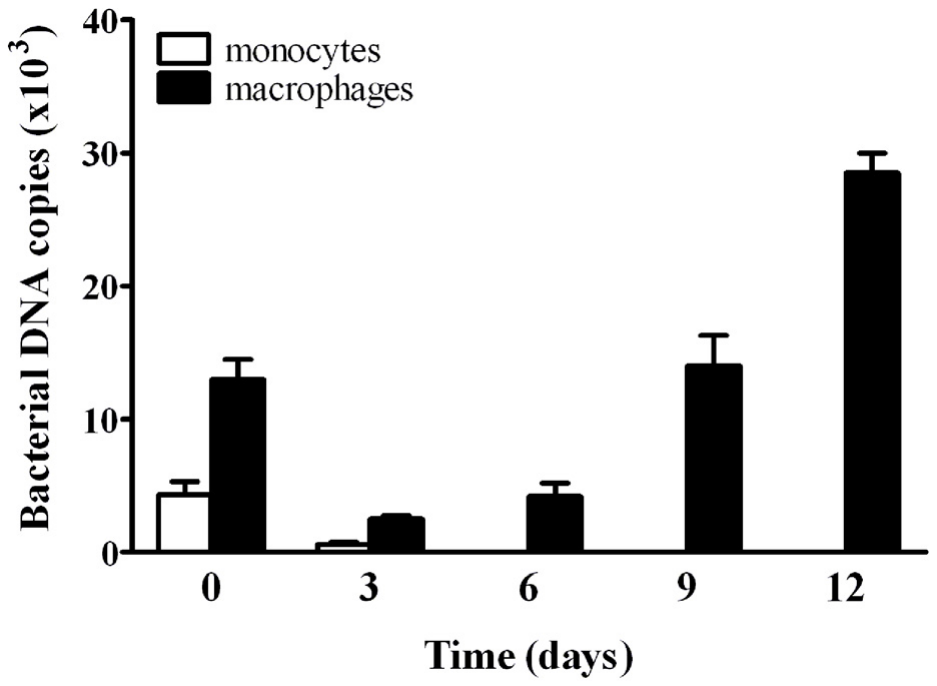
*T. whipplei* replicates in macrophages but not in monocytes. Monocytes and macrophages were incubated with *T. whipplei* for four hours (50 bacteria/cell), washed to discard unphagocytosed bacteria and incubated for different time periods. Bacterial uptake and replication were assessed by determining the bacterial DNA copy number by qPCR. The results are expressed as the mean ± SEM of four experiments performed in triplicate.

In the first series of experiments, human monocytes and macrophages were infected with *T. whipplei* (50 bacteria/cell) for four hours (designated as day 0) and washed to discard unphagocytosed bacteria. The intracellular fate of *T. whipplei* was assessed for 12 days. As published before [6], monocytes eliminated *T. whipplei*, whereas *T. whipplei* replicated within macrophages after a transient phase of *T. whipplei* elimination (**Figure 1**).

**Figure 2:**
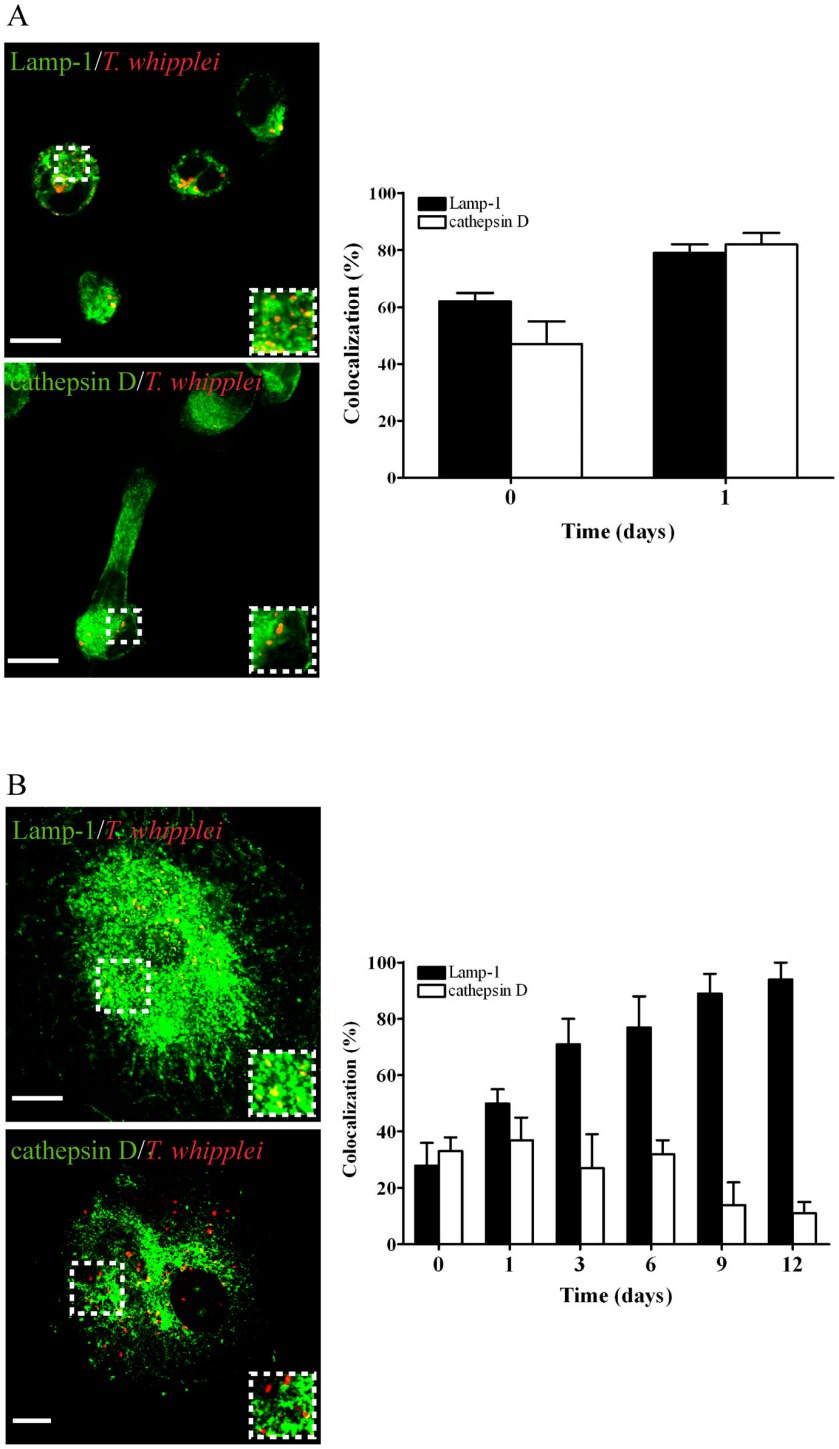
*T. whipplei* localization in monocytes and macrophages. The intracellular localization of *T. whipplei* within monocytes at day 1 (**A**) and macrophages at day 12 (**B**) was assessed by indirect immunofluorescence and laser scanning microscopy. Scale bars represent 5 µm. The percentages of *T. whipplei* that colocalized with cathepsin D or Lamp-1 in monocytes (**A**) and macrophages (**B**) were determined. More than 300 phagosomes were examined per experimental condition, and the results are expressed as the mean ± SEM of three independent experiments.

We next studied the nature of the *T. whipplei*-containing compartments in both monocytes and macrophages. In monocytes, *T. whipplei* was eliminated within phagolysosomes that acquired both Lamp-1 and cathepsin D. Specifically, 62±3% of *T. whipplei* phagosomes colocalized with Lamp-1, and 47±8% colocalized with cathepsin D at day 0. These percentages increased to 80% at day 1, and intact bacteria were no longer detected thereafter (**Figure 2A**). In macrophages, bacteria were located in late phagosomes. Specifically, 28±8% of *T. whipplei* phagosomes were positive for Lamp-1 at day 0; this percentage increased to 71±9% and 94±6% after 3 and 12 days, respectively. In a first phase, *T. whipplei* colocalized with cathepsin D (**Figure 2B**): 33±5% of bacteria colocalized with cathepsin D at day 0, and 37±8% colocalized with it at day 1, suggesting that bacteria were transiently eliminated within phagolysosomes. This phenomenon might be related to the dramatic decrease in the *T. whipplei* DNA copy number observed between day 0 and 3 (**Figure 1**). In a second phase (day 3–12), the percentage of *T. whipplei* colocalizing with cathepsin D steadily decreased: at day 12, it was only 11±4% (**Figure 2B**). This phenomenon might be related to the increase in the *T. whipplei* DNA copy number observed between days 3 and 12 (**Figure 1**). Taken together, these results suggest that *T. whipplei* replication in macrophages is associated with the presence of bacteria within late phagosomes that are unable to fuse with lysosomes.

### Effect of IL-16 on *T. whipplei* localization

**Figure 3:**
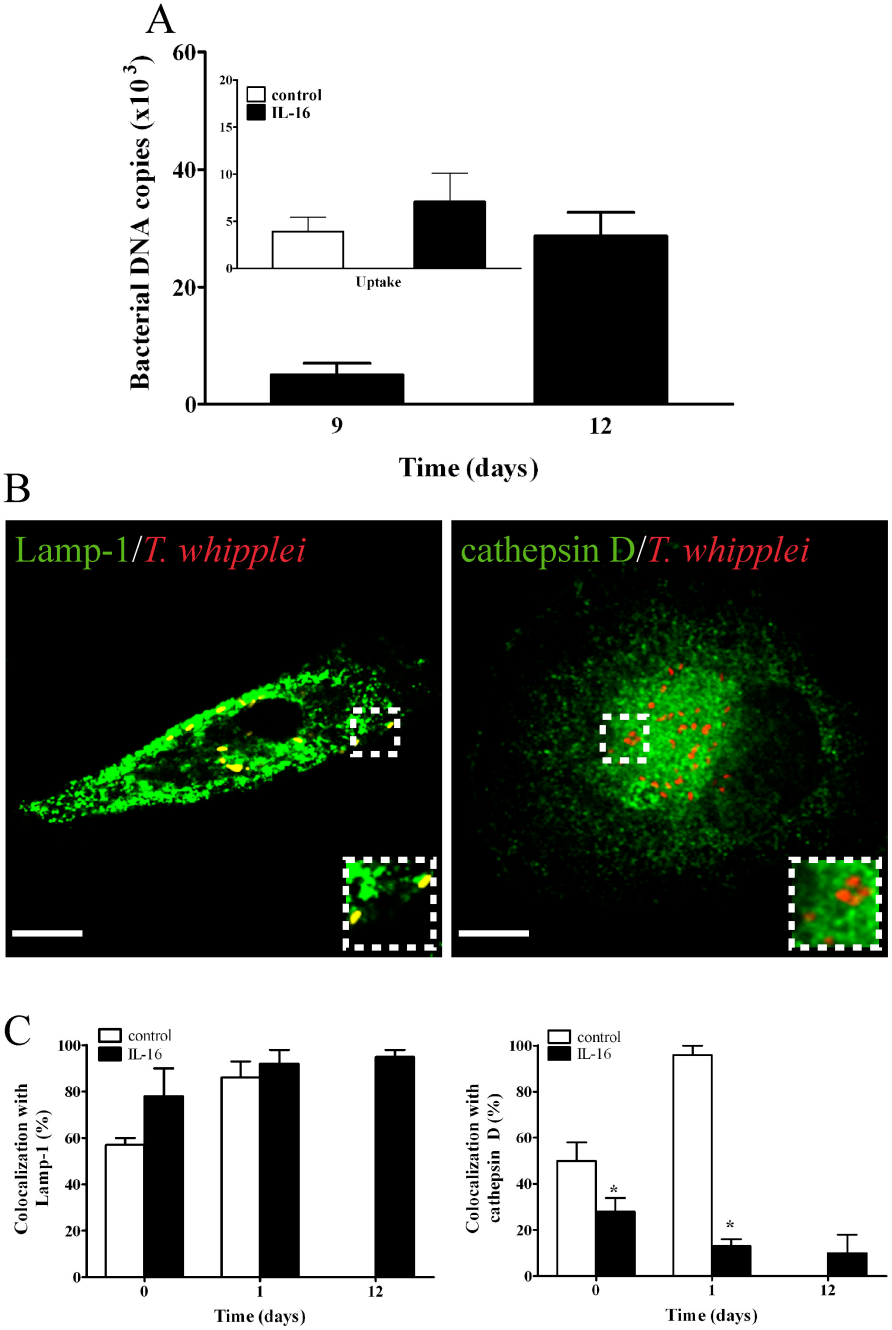
Effect of exogenous IL-16 on *T. whipplei* fate in monocytes. Monocytes were treated with or without IL-16 for 18 hours, incubated with *T. whipplei* for four hours (50 bacteria/cell), washed to remove unphagocytosed bacteria and incubated for additional time periods. (**A**) *T. whipplei* uptake (**inset**) and replication were assessed by determining the bacterial DNA copy number by qPCR after 9 and 12 days of infection. The results are expressed as the mean ± SEM of four experiments performed in triplicate. (**B**) The intracellular localization of *T. whipplei* within IL-16-treated monocytes was assessed by indirect immunofluorescence and laser scanning microscopy at day 12. Scale bars represent 5 µm. (**C**) The percentages of *T. whipplei* that colocalized with cathepsin D or Lamp-1, respectively, were determined (*n* = 5). More than 300 phagosomes were examined per experimental condition, and the results are expressed as the mean ± SEM of five independent experiments. *p*<0.05.

**Figure 4:**
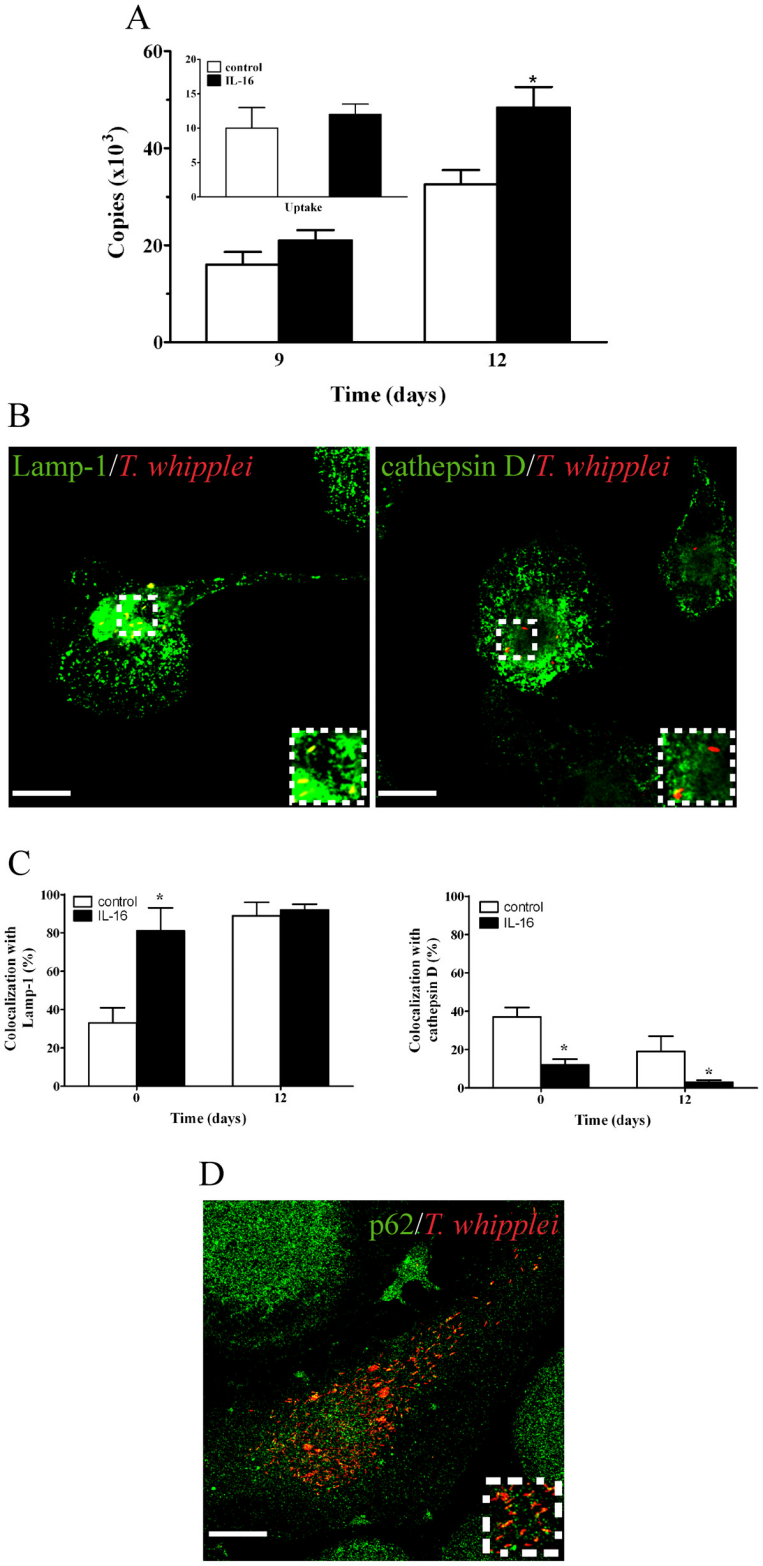
Effect of exogenous IL-16 on *T. whipplei* fate in macrophages. Macrophages were treated with rhIL-16 as described in Figure 3. (**A**) *T. whipplei* uptake (**inset**) and replication were determined by qPCR. The results are expressed as the mean ± SEM of four independent experiments performed in triplicate. (**B**) The intracellular localization of *T. whipplei* was analyzed by indirect immunofluorescence and laser scanning microscopy at day 12. Scale bars represent 5 µm. (**C**) The percentage of organisms that colocalized with Lamp-1 or cathepsin D, respectively, was determined. More than 300 phagosomes were examined per experimental condition, and the results are expressed as the mean ± SEM of four independent experiments. *p*<0.05. (**D**) The localization of organisms with p62, a specific marker for autophagosomes, was assessed by indirect immunofluorescence and laser scanning microscopy. More than 200 phagosomes were examined in two independent experiments.

Given that IL-16 release is related to *T. whipplei* replication [6], we asked if exogenous IL-16 inhibits the conversion of *T. whipplei* phagosomes to phagolysosomes. To address this question, monocytes were treated for 16 hours with 10 ng/ml of recombinant human (rh) IL-16 prior to *T. whipplei* infection [6]. The pretreatment of monocytes with IL-16 did not significantly alter bacterial uptake (**Figure 3A** **, inset**). *T. whipplei* replication occurred to an extent similar to that observed in macrophages **(comparison between** **Figure 3A** **and** **Figure 1** **)** as published before [6]. However, *T. whipplei* colocalized with Lamp-1 but not with cathepsin D in IL-16-treated monocytes (**Figure 3B**). The percentage of phagosomes containing *T. whipplei* colocalizing with cathepsin D fell significantly (*p*<0.05) (two-fold) between days 0 and 1. At day 12, *T. whipplei* was only present in IL-16-treated cells, in which it resided in phagosomes associated with Lamp-1, but not with cathepsin D (**Figure 3C**). We also studied the effect of exogenous IL-16 on the intracellular fate of *T. whipplei* in macrophages. IL-16 did not affect *T. whipplei* uptake but it significantly (*p*<0.05) increased bacterial replication (**Figure 4A**) as published before [6]. It also inhibited the acquisition of cathepsin D by *T. whipplei* phagosomes (**Figure 4** **, B and C**). Next, we wondered whether IL-16 re-routed *T. whipplei* phagosomes towards the autophagosome pathway. We found that Ab directed against p62, a marker for autophagosomes [35], did not colocalize with *T. whipplei* (**Figure 4D**). Finally, we investigated if the effect of IL-16 on *T. whipplei* trafficking was specific using latex beads. Latex beads were located within phagolysosomes at days 1 and 12 post-ingestion. IL-16 did not modify the latex beads localization with either of the two markers (**Figure S1**), demonstrating that IL-16 specifically acted on *T. whipplei* trafficking. Taken together, these data show that incubating monocytes or macrophages with exogenous IL-16 can increase *T. whipplei* replication within phagosomes that are unable to be converted to phagolysosomes.

### Inhibition or absence of IL-16 rescued the maturation of *T. whipplei* phagosomes

**Figure 5:**
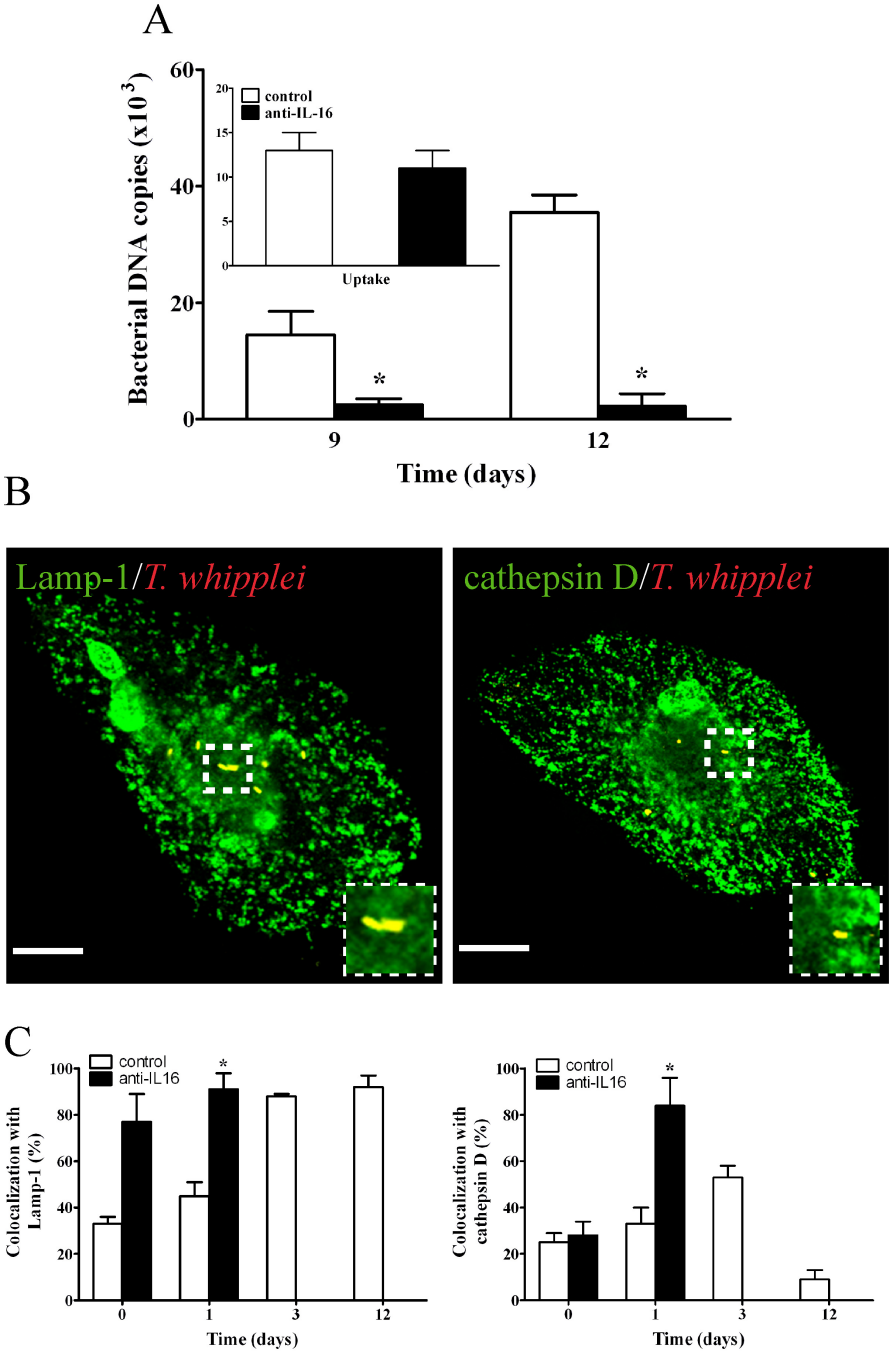
Effect of anti-IL-16 Abs on *T. whipplei* fate in macrophages. Macrophages were treated with or without anti-IL-16 blocking Abs for 18 hours, incubated with *T. whipplei* for 4 hours, washed to remove unphagocytosed bacteria and incubated for additional time periods in the presence of blocking Abs. (**A**) *T. whipplei* uptake (**inset**) and replication were assessed by determining the bacterial DNA copy number by qPCR. The results are expressed as the mean ± SEM of four independent experiments performed in triplicate. (**B**) The intracellular localization of *T. whipplei* within macrophages treated with anti-IL-16 Abs was assessed by indirect immunofluorescence and laser scanning microscopy at day 1. Scale bars represent 5 µm. (**C**) The percentage of *T. whipplei* that colocalized with cathepsin D or Lamp-1, respectively, was determined. More than 300 phagosomes were examined per experimental condition, and the results are expressed as the mean ± SEM of three independent experiments. *p*<0.05.

**Figure 6:**
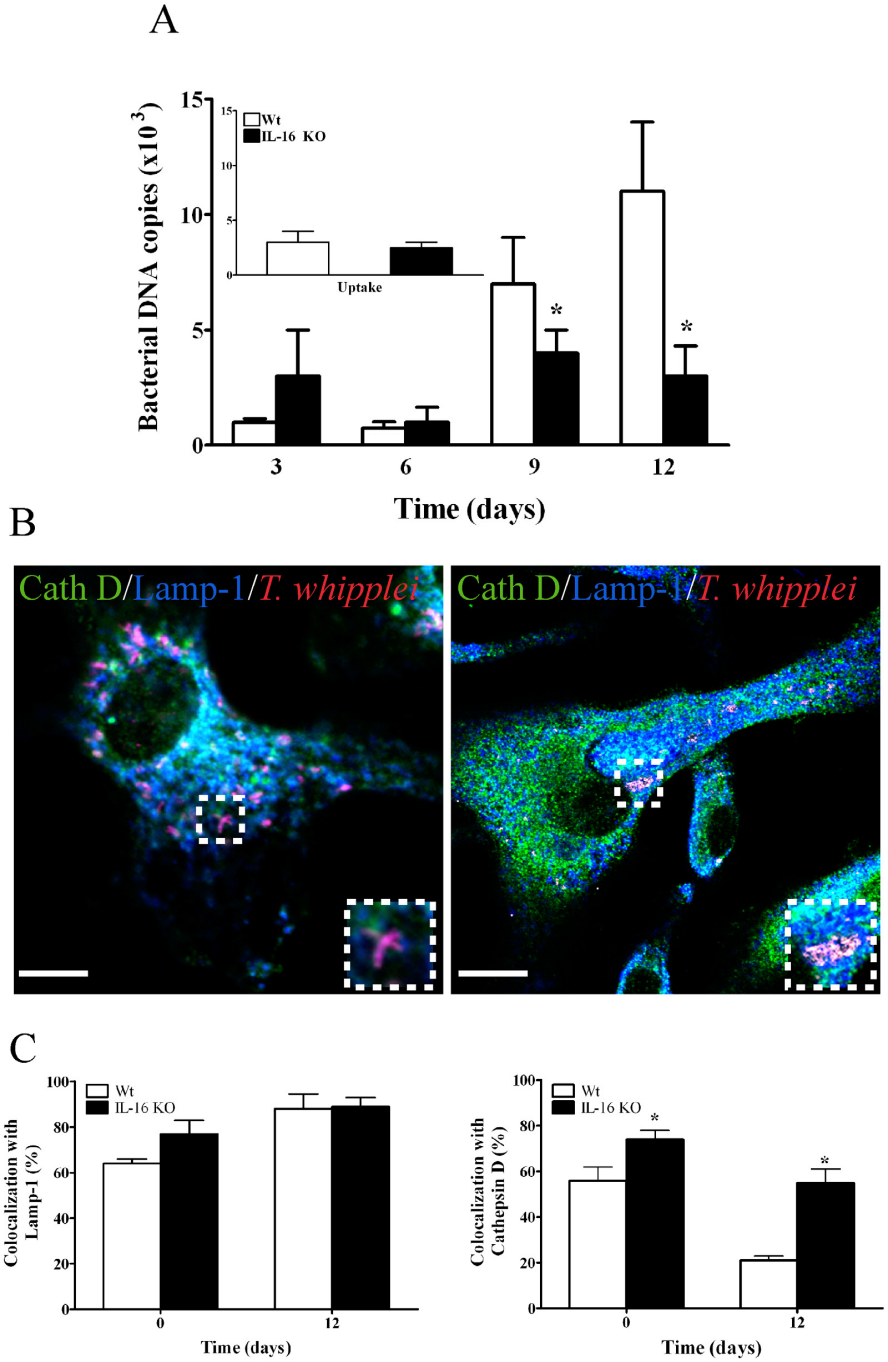
*T. whipplei* fate in IL-16^−/−^ BMDM. BMDMs from wt and IL-16^−/−^ mice were infected with *T. whipplei* similarly to the infection of human macrophages. (**A**) *T. whipplei* uptake (inset) and replication were determined by qPCR. The results are expressed as the mean ± SEM of four independent experiments performed in triplicate. (B) The intracellular localization of *T. whipplei* within wt (left panel) and IL-16^−/−^ (right panel) BMDMs was assessed by indirect immunofluorescence and laser scanning microscopy at day 12. Scale bars represent 5 µm. (**C**) The percentage of *T. whipplei* that colocalized with Lamp-1 or cathepsin D, respectively, was determined. More than 300 phagosomes were examined per experimental condition, and the results are expressed as the mean ± SEM of three independent experiments. The colocalization of *T. whipplei* (red) with cathepsin D (green) appears as yellow; the colocalization of *T. whipplei* with Lamp-1 (blue) appears as purple; and the colocalization of *T. whipplei* with Lamp-1/cathepsin D appears as white. *p*<0.05.

We then investigated the effect of IL-16 inhibition on the replication and localization of *T. whipplei* in macrophages. Treatment of macrophages with anti-IL-16 antibodies (Abs) did not alter *T. whipplei* uptake but it dramatically inhibited bacterial replication (**Figure 5A**) as published before [6]. It also induced the conversion of *T. whipplei* phagosomes to phagolysosomes (**Figure 5B**). Specifically, about 80% of *T. whipplei* phagosomes colocalized with Lamp-1 and cathepsin D at day 1, and bacteria were no longer detected thereafter (**Figure 5C**). Hence, IL-16 was required to maintain *T. whipplei* localization within late phagosomes. Second, the intracellular fate and localization of *T. whipplei* were studied in bone marrow-derived macrophages (BMDM) from IL16^−/−^ mice. *T. whipplei* uptake by IL-16^−/−^ and wild type (wt) BMDMs was similar (**Figure 6A** **, inset**). In wt BMDMs, bacterial replication was intense at days 9 and 12 post-infection (**Figure 6A**) as published before [36] and similar to that observed in human macrophages (**see** **Figure 1**). In IL-16^−/−^ BMDMs, the bacterial DNA copy number was 3.5 times lower (*p*<0.05) than that in wt BMDMs at days 9 and 12 (**Figure 6A**), demonstrating that *T. whipplei* replication was inhibited in the absence of IL-16. This phenomenon was related to *T. whipplei* colocalization with phagolysosomes. Specifically, in wt and IL-16^−/−^ BMDMs, the percentage of *T. whipplei* phagosomes that colocalized with cathepsin D was higher in IL-16^−/−^ BMDMs than in wt BMDMs (74±4% vs. 56±6% at day 0, *p*<0.05; 55±6% vs. 21±2% at day 12, *p*<0.04) (**Figure 6B and 6C**). Taken together, these results demonstrate that the microbicidal competence of macrophages towards *T. whipplei* can be restored in the absence of IL-16.

### Effect of IFNγ on *T. whipplei* intracellular localization and IL-16 production

**Figure 7:**
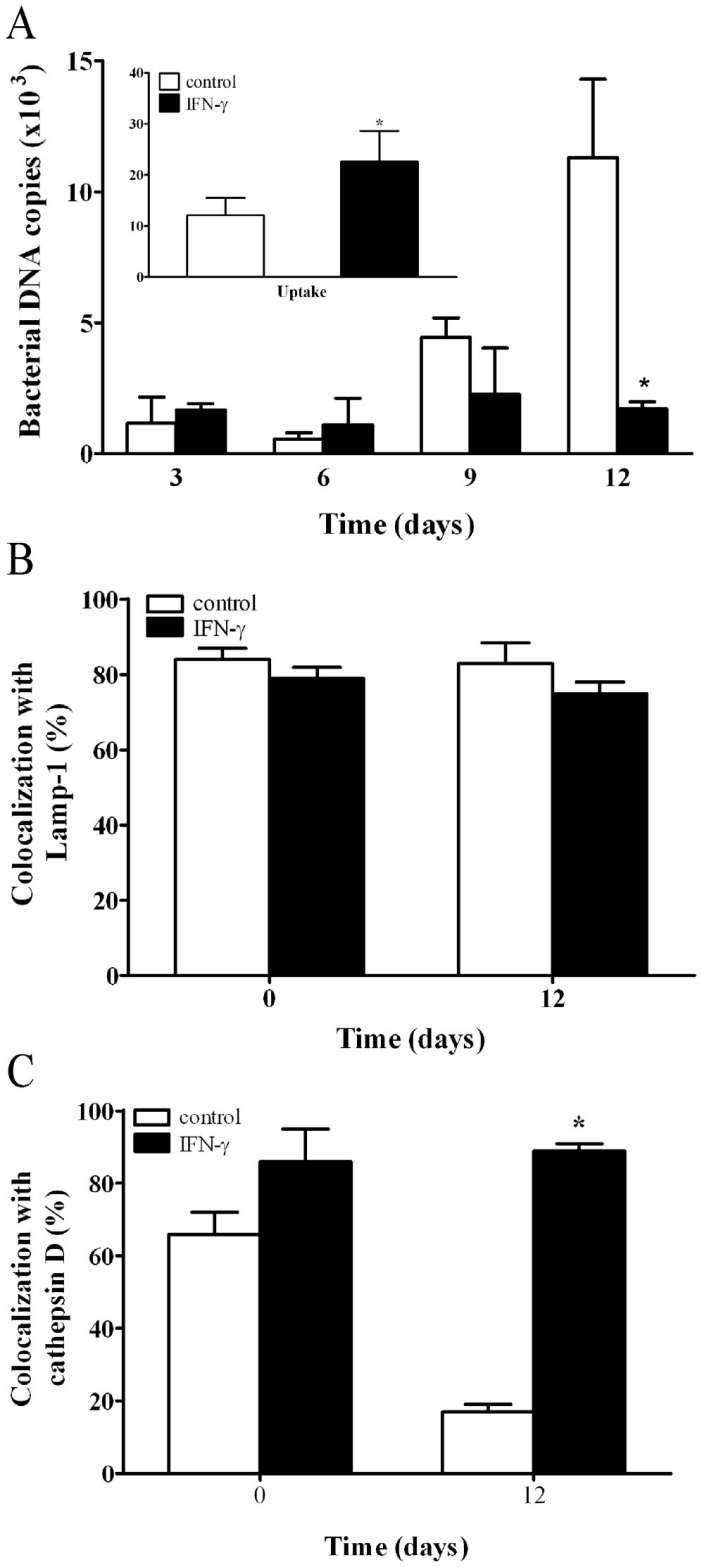
Effect of exogenous IFNγ on *T. whipplei* fate in macrophages. Macrophages were treated with rhIFNγ (500 UI/ml) and then infected with *T. whipplei* (50 bacteria/cell). (**A**) *T. whipplei* uptake (**inset**) and replication were determined by qPCR. The results are expressed as the mean ± SEM of four independent experiments performed in triplicate. (**B, C**) The intracellular localization of *T. whipplei* within IFNγ-treated macrophages was assessed by indirect immunofluorescence and laser scanning microscopy. The percentage of *T. whipplei* that colocalized with Lamp-1 (**B**) or cathepsin D (**C**) was determined. More than 300 phagosomes were examined per experimental condition, and the results are expressed as the mean ± SEM of five independent experiments. *p*<0.05.

Given that the absence of IL-16 is associated with *T. whipplei* elimination by macrophages, and that impaired IFNγ production may be a cause of the delayed *T. whipplei* elimination in WD patients [4], we looked for a connection between IL-16 and IFNγ in *T. whipplei* infection. To this end, macrophages were treated for 16 hours with recombinant IFNγ prior to *T. whipplei* infection. IFNγ-treated macrophages internalized *T. whipplei* more efficiently than untreated macrophages (*p*<0.05), but *T. whipplei* replication was abolished at day 12 (*p*<0.05) (**Figure 7A**). The colocalization of *T. whipplei* with Lamp-1 in untreated and IFNγ-treated macrophages was close to 80% at day 0 and did not change thereafter (**Figure 7B**). In contrast, while the percentage of *T. whipplei* colocalizing with cathepsin D decreased from 66±6% to 17±2% after 12 days in untreated cells, more than 85% of *T. whipplei* phagosomes colocalized with cathepsin D in IFNγ-treated macrophages (**Figure 7C**). We then asked if IFNγ stimulates *T. whipplei* elimination by macrophages by interfering with IL-16 production. IL-16 production induced by *T. whipplei* was completely inhibited when macrophages were pretreated with IFNγ (**Table 1**). Furthermore, IFNγ inhibited the induction of *T. whipplei* replication by IL-16 (**Table 2**). These data clearly show that IFNγ regulates IL-16 production, and IL-16 in turn mediates *T. whipplei* replication.

**Table 1:** Effect of IFNγ on IL-16 secretion^a^.

IFNγ	−		+	
*T. whipplei*	−	+	−	+
IL-16 secretion	8.2±5	700±101	<6.2	<6.2

a Macrophages were pre-treated with or without rhIFNγ (500 UI/ml) for 16 hours and then infected with *T. whipplei* (50 bacteria/cell). Supernatants were collected after 48 hours and assayed for the presence of IL-16 by immunoassay. The results are expressed as the mean ± SEM (pg/ml) of three independent experiments performed in triplicate.

**Table 2:** Effect of IFNγ on IL-16-stimulated replication of *T. whipplei* a.

	control	IL-16	IL-16+IFNγ
*T. whipplei* DNA copies (×10^3^)	28.6±2.1	63.4±5.5	0

a Macrophages were pretreated with or without rhIL-16 (10 ng/ml) for 16 hours, infected with *T. whipplei* (50 bacteria/cell) in the absence or presence of rhIFNγ, washed to discard unphagocytosed bacteria and incubated for nine days in the absence or presence of IFNγ. Bacterial DNA copies were quantified by qPCR at day 9. The results are expressed as the mean ± SEM of four independent experiments performed in triplicate.

### Microarray analysis of IL-16^−/−^ macrophages stimulated with *T. whipplei*

**Figure 8:**
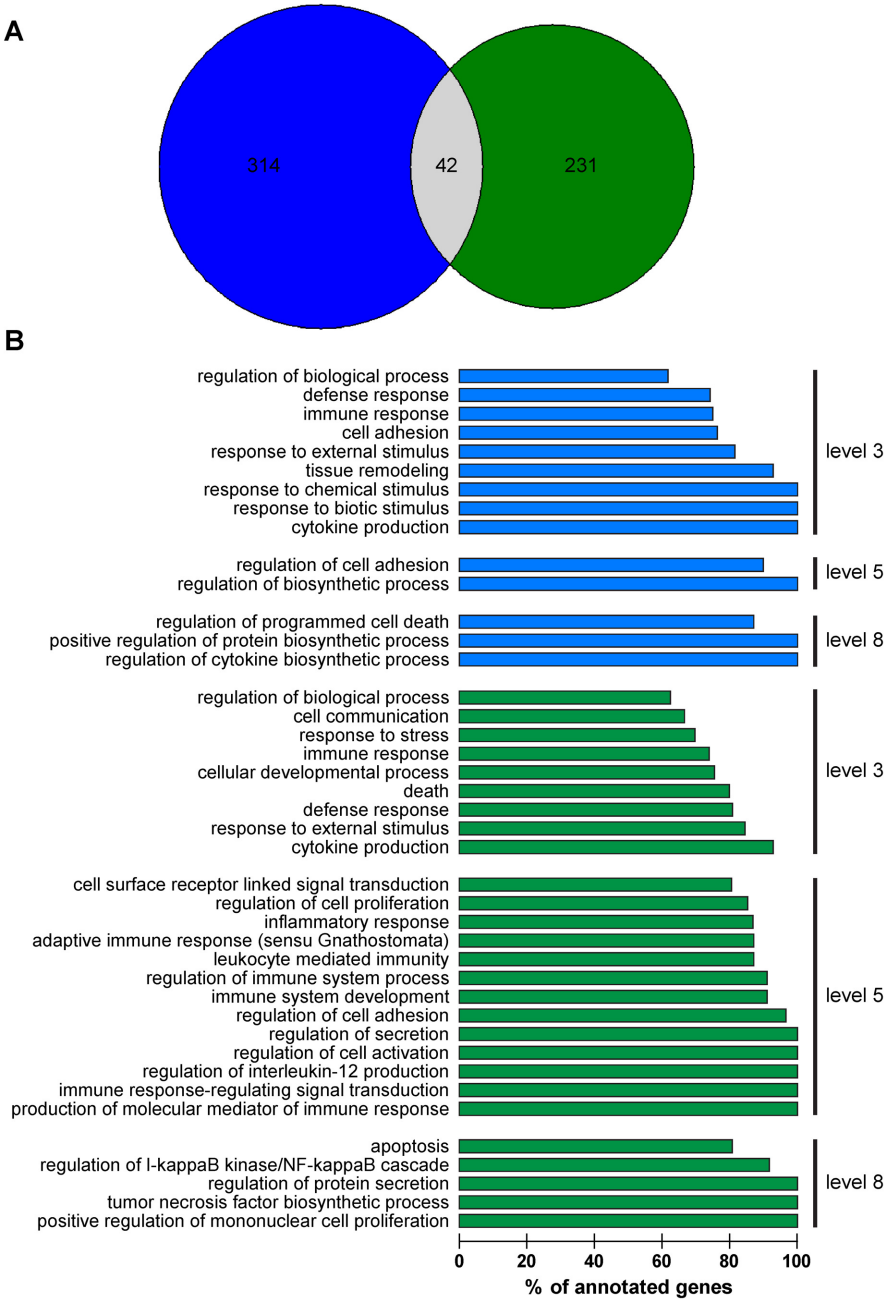
Analysis of transcriptional responses of BMDMs to *T. whipplei.* BMDMs were stimulated with *T. whipplei* (50 bacteria/cell) for six hours and host responses were analyzed using whole genome microarrays. (**A**) Significant features were compared between wt (blue) and IL-16^−/−^ (green) BMDMs and represented by a Venn diagram. Common significant features are displayed in grey. (**B**) Significantly over-represented GO biological processes in *T. whipplei*-stimulated wt (blue) and IL-16^−/−^ (green) BMDM were determined by applying the two-tailed Fisher's exact test (*p*<0.05).

**Figure 9:**
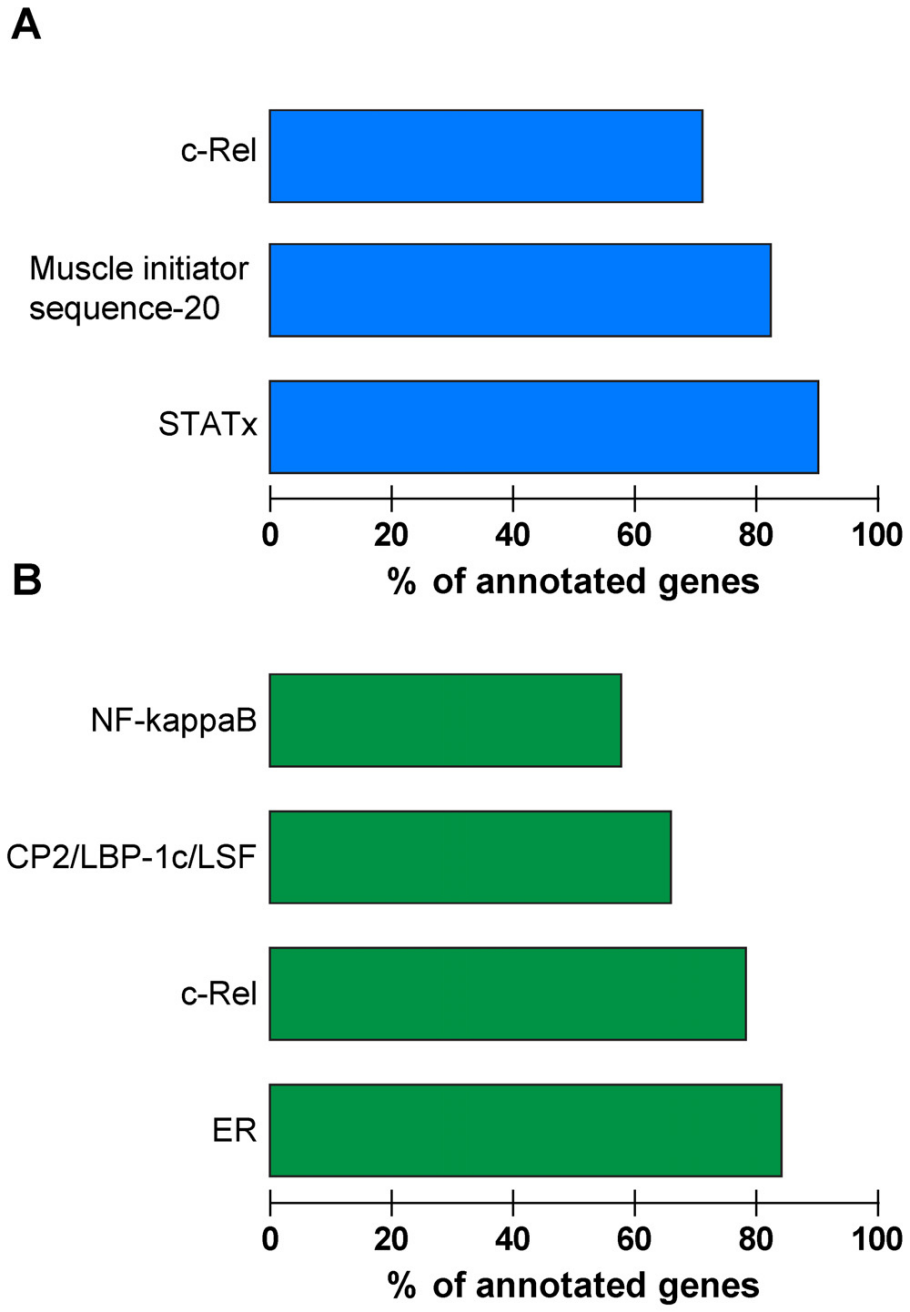
Transcription factors activated by *T. whipplei* in BMDMs. BMDMs were stimulated with *T. whipplei* for six hours and host responses were analyzed using whole genome microarrays. Significantly over-represented transcription factors involved in the transcriptional responses of *T. whipplei*-stimulated wt (**A**) and IL-16^−/−^ (**B**) BMDMs were determined using the Fatiscan tool.

As wt BMDMs infected with *T. whipplei* have been shown to display an atypical activation program combining M2 polarization, a type I IFN response and apoptosis [36], host responses towards *T. whipplei* in IL-16^−/−^ BMDM were monitored using a full genome microarray, and data were compared to those previously reported [36] (GEO database at NCBI, accession number GSE16180). After a six-hour stimulation with *T. whipplei*, 356 and 273 probes were significantly modulated in wt and IL-16^−/−^ BMDMs, respectively. Among them, only 42 probes were similarly modulated in both wt and IL-16^−/−^ BMDMs (**Figure 8A** **and Table S1**). Next, the genes were annotated according to functional classes. Gene Ontology (GO) biological processes at level 3 did not detect major differences between wt and IL-16^−/−^ BMDM responses to *T. whipplei* (**Figure 8B**). Indeed, the GO terms of immune response (GO: 0006955), defense response (GO: 0006952), response to external stimulus (GO: 0009605), regulation of biological process (GO: 0050789) and cytokine production (GO: 0001816) were significantly over-represented in both wt and IL-16^−/−^ BMDMs. In contrast, when GO biological processes were analyzed at lower levels, functional differences were identified. For example, analysis of GO biological processes at level 5 revealed 2 over-represented GO terms in wt BMDMs, different from the 13 over-represented GO terms in IL-16^−/−^ BMDMs. Importantly, among these 13 GO terms, 10 were linked to immune response (**Figure 8B**). A closer analysis of GO biological processes at level 8 revealed that the regulation of the I-κB kinase/NF-κB cascade (GO: 0043122) was over-represented in IL-16^−/−^ BMDMs but not in their wt counterparts (**Figure 8B**). These observations were further confirmed by an analysis of over-represented genes according to their transcription factors. Specifically, c-Rel and STAT were over-represented in wt BMDMs (**Figure 9A**), in accordance with recent data demonstrating that *T. whipplei* induces type I IFN-dependent responses in these cells [36]. In contrast, in IL-16^−/−^ BMDMs, the main transcription factors regulated by *T. whipplei* were NF-κB, CP2/LBP-1C/LSF and c-Rel (**Figure 9B**), all involved in inflammatory responses. Taken together, these results show that the activation programs induced by *T. whipplei* in wt and IL-16^−/−^ BMDMs are different, and that IL-16 might be involved in macrophage activation. This effect was specific because no significant terms were found between wt and IL-16^−/−^ BMDMs stimulated with lipolysaccharide (LPS). Using the FatiGO Compare tool, 11,235 and 12,107 features were significantly modulated in wt and IL-16^−/−^ BMDMs, respectively. The large majority (9121) of them were common to both wt and IL-16^−/−^ BMDMs (**Figure S2**).

## Discussion

We describe here a previously unreported ability of IL-16 to modulate anti-bacterial defenses. IL-16 inhibited *T. whipplei* killing by inhibiting the maturation of bacterial phagosomes into phagolysosomes. The intracellular fate of *T. whipplei* differed dramatically between monocytes and macrophages. In monocytes, *T. whipplei* was rapidly eliminated in phagolysosomes characterized by the presence of Lamp-1 and cathepsin D. In macrophages, a large number of bacteria were eliminated in an early phase, but the remaining bacteria survived and intensively replicated in a late phase. Similar results were obtained in a *Brucella* infection model, in which the majority of bacteria were eliminated, while only few bacteria survived and then replicated within a suitable compartment [37]. When *T. whipplei* organisms replicated, they were localized in late phagosomes harboring Lamp-1 but devoid of cathepsin D, suggesting a blockage of phagosome-lysosome fusion.

We have also found that IL-16 inhibits the ability of both human monocytes and macrophages to eliminate *T. whipplei*. First, adding exogenous IL-16 to monocytes induced *T*. *whipplei* replication and increased bacterial replication in macrophages as published before [6]. Second, blocking IL-16 Abs induced *T. whipplei* elimination as published before [6]. Third, *T. whipplei* replicated in murine BMDMs, whereas bacterial replication was inhibited in BMDMs from IL-16^−/−^ mice. The effect of IL-16 on the microbicidal responses of monocytes and macrophages cannot be attributed to a deactivation effect similar to that induced by IL-10 known to stimulate *C. burnetii* replication within macrophages [38]. Indeed, IL-10 was unable to induce *T. whipplei* replication in monocytes or to increase *T. whipplei* replication in macrophages (**Figure S3**). On the other hand, the effect of IL-16 on *T. whipplei* replication was specific since IL-16 had no effect on *C. burnetii* replication (**Figure S4**). These results indicate that IL-16 specifically favors *T. whipplei* replication.

The inhibition of *T. whipplei* elimination by IL-16 was likely related to the inhibited phagosome-lysosome fusion. In IL-16-treated monocytes and macrophages, *T. whipplei* resided in late phagosomes unable to fuse with lysosomes. Blocking IL-16 by specific Abs led to *T. whipplei* elimination within phagolysosomes. In mouse BMDMs that did not express IL-16, *T. whipplei* were also eliminated in phagolysosomes. The effect of IL-16 on the maturation of *T. whipplei* phagosomes was specific because IL-10 did not affect *T. whipplei* localization within phagolysosomes in monocytes or that within late phagosomes in macrophages (**Figure S5**). IL-10 has been previously demonstrated to favor the localization of mycobacteria within early phagosomes [29] and to increase the capacity of *C. burnetii* to traffic into a non-mature late phagosome [32], [33]. In addition, IL-16 specifically controlled *T. whipplei* phagosome conversion because IL-16 had no effect on latex bead and *C. burnetii* (**Figure S4**) trafficking. Cytokines such as IFNγ, IL-4 and IL-13 have been described to induce or inhibit autophagy. Specifically, IFNγ has been shown to re-route the phagosome conversion pathway towards the autophagy pathway [39]. We demonstrated that the effect of IL-16 on the intracellular localization of *T. whipplei* was not associated with a re-routing of the phagosome conversion pathway towards the autophagosome pathway because p62, a marker for autophagosomes [35], did not colocalize with *T. whipplei.*

The mechanisms elicited by IL-16 that enable *T. whipplei* replication by blocking the late endosome stage were not related to defective recruitment of several molecules involved in phagosome conversion and phagolysosome biogenesis. It has been demonstrated that cytokines such as IL-6 and IL-12 specifically affect the expression of the GTPases Rab5 and Rab7, which are essential for membrane trafficking events leading to phagosome-lysosome fusion [40]. IFNγ also selectively induces Rab5a synthesis [41]. We showed here that IL-16 did not affect the transcription of either Rab5 or Rab7 (**Figure S6**). IL-16 might also inhibit the fusion of *T. whipplei* phagosomes with lysosomes by modulating the protein level of cathepsin D present on macrophages. Our results clearly showed that IL-16 did not affect the protein expression of cathepsin D or Lamp-1 (**Figure S6**). IFNγ has been demonstrated to affect the abundance, recruitment and phosphorylation of several proteins involved in phagosome conversion [28] and to affect Rab prenylation [41]. We therefore hypothesize that IL-16 might also affect post-transduction events in molecules engaged in phagolysosome biogenesis. Note that cathepsin D and Lamp-1 arrive at lysosomes via different trafficking routes. Lamp-1 is delivered to late endosomes and concentrates in lysosomes whereas cathepsin D is delivered to late endosomes via the mannose-6-phosphate receptor. We cannot exclude that IL-16 affects specifically one of these routes. An another hypothesis is that IL-16 acts on *T. whipplei* replication and phago-lysosome fusion by favoring a faster maturation of monocytes into macrophages since IL-16 has been described to accelerate differentiation of monocytes into macrophages [6].

Although IL-16 is considered a Th1-related cytokine [18], our data suggest that IL-16 may also behave as a Th2-related cytokine. In accordance with this hypothesis, IL-16 has been shown to activate Stat-6, which is known to play a role in the polarization of CD4^+^ T cells to the Th2 phenotype [42]. Consequently, we assumed that an imbalance between IL-16 and IFNγ was likely to occur in our case. First, IFNγ induced both the elimination of *T. whipplei* by macrophages in a dose-dependent manner (**Table S2**) and phagosome conversion to phagolysosomes. Second, IFNγ inhibited both IL-16 production by macrophages and IL-16-stimulated *T. whipplei* replication. It is likely that IFNγ stimulates the microbicidal response of macrophages towards *T. whipplei* by impairing the IL-16 pathway.

To further investigate the downstream effectors of IL-16, we analyzed the transcriptomic response to *T. whipplei* in IL-16^−/−^ macrophages using a whole genome array and previouly reported data [36]. In the absence of IL-16, the expression of 273 probes was modulated by *T. whipplei*. The great majority of these probes were not affected by *T. whipplei* in macrophages expressing IL-16. This response was different from that to LPS treatment, in which most modulated genes were common to macrophages with and without IL-16 expression. The analysis of GO biological processes revealed the enrichment of 13 GO terms linked to immune response. Specifically, there was over-representation of the I-κB kinase/NF-κB cascade. This finding is in agreement with our recent publication in which the activation of transduction pathways by *T. whipplei* involves a detrimental type I IFN signaling pathway and a poor NF-κB response [36]. In the absence of IL-16, we speculate that the activation of macrophages by *T. whipplei* might favor the NF-κB pathway instead of the type 1 IFN pathway. As a consequence, we can assume that IL-16 does not polarize macrophages toward M2 cells. First, only genes from the NF-κB pathway were modulated in IL-16^−/−^ BMDMs stimulated with *T. whipplei* suggesting a partial effect of IL-6 on macrophages polarization. Second, the effect of IL-16 on *T. whipplei*-stimulated response was specific because the transcriptional response induced by LPS were similar in wt and IL-16^−/−^ BMDMs.

In conclusion, this study demonstrates for the first time that IL-16 induces *T. whipplei* replication by blocking the normal conversion of *T. whipplei* phagosomes into phagolysosomes. Our data also suggest that IL-16 inhibits the fusion of *T. whipplei* phagosomes with lysosomes by inducing a macrophage activation program that causes the IFNγ and NF-κB pathways to become essential to the elimination of *T. whipplei*. These results might explain why IFNγ was efficient for the treatment of refractory WD. They also suggest that IL-16 blockade by IFNγ, specific Abs or RNAi might contribute to the therapeutic elimination of *T. whipplei* in WD patients.

## Methods

### *T. whipplei* culture

The *Twist*-Marseille strain of *T. whipplei* (CNCM I-2202) was cocultured with HEL cells (CCL-37; American Type Culture Collection) and purified as previously described [34]. Bacteria were counted by Gimenez staining and indirect immunofluorescence, and their viability was assessed using the LIVE/DEAD BacLight Bacterial Viability Kit (Molecular Probes).

### Cell culture

Peripheral blood mononuclear cells (PBMCs) were isolated from leukopacks (Etablissement Français du Sang) by Ficoll gradient (MSL, Eurobio). Monocytes were then isolated using CD14^+^ columns as recommended by the manufacturer (Miltenyi Biotec). More than 95% of the cells were CD14^+^ monocytes as determined by flow cytometry. Macrophages were derived from monocytes by a seven-day culture in RPMI 1640 containing human AB serum: more than 95% of cells were considered as macrophages since they expressed CD68 as determined by flow cytometry. Monocytes and macrophages (10^5^ cells/assay) were incubated in flat-bottom 24-well plates containing glass coverslips for immunofluorescence experiments. Then, they were infected with *T. whipplei* for four hours, washed to remove unphagocytosed bacteria and incubated for designated periods in RPMI 1640 containing 10% fetal calf serum (FCS) [6]. In some experiments, rhIL-16 (10 ng/ml) [6], rhIL-10 (10 ng/ml) or rhIFNγ (500 UI/ml) (R&D Systems) was added to monocytes and macrophages 18 hours prior to infection. Endogenous IL-16 was neutralized using 1 µg/ml anti-IL-16 mAbs (R&D Systems) as previously described [6].

BMDMs were generated from 6- to 8-week-old IL-16^−/−^ C57BL/6 and littermate controls, as previously described [43]. Briefly, mice were euthanized by cervical dislocation, and bone marrow was flushed out from femurs and tibias. Bone marrow progenitors were seeded in Petri dishes in RPMI supplemented with 10% FCS, 2 mM L-glutamine, 100 UI/ml penicillin and 100 µg/ml streptomycin supplemented with 15% L929 cell supernatant and allowed to differentiate for seven days [44]. Differentiated BMDMs were seeded (10^5^ cells/well containing a glass coverslip for immunofluorescence analysis) in 24-well tissue culture plates in RPMI medium.

### Ethics Statement

All animal experiments followed the guiding principles of animal care and use defined by the Conseil Scientifique du Centre de Formation et de Recherche Experimental Médico-Chirurgical (CFREMC) with the rules of Décret N° 87-848 of 10/19/1987 and were approved by the ethics board of the university at which the experiments were performed (Faculté de Médecine de la Timone, Experimentation permit number 13.385).

### Quantitative real-time PCR (qPCR)

Cells were lyzed, and DNA was extracted using the QIAamp DNA Mini Kit (Qiagen). PCR was performed using the LightCycler-FastStart DNA Master SYBR Green system (Roche) with primers specific for the *T. whipplei* 16S-23S ribosomal intergenic spacer region (tws3f and tws4r) as previously described [45]. The sequences of primers for *T. whipplei* were as follows: ccggtgacttaacctttttggaga (left primer) and tcccgaggcttatcgcagattg (right primer). In each PCR run, a standard curve was generated using serial dilutions ranging from 10 to 10^8^ copies of the intergenic spacer region, and the results were calculated using the LightCycler 5.32 software (LC-Run version 5.32, Roche).

### Colocalization experiments

Cells (10^5^ cells/assay) were infected with *T. whipplei* (50 bacteria/cell) for four hours, extensively washed to discard unphagocytosed bacteria and then incubated for different time periods before fixation in 3% paraformaldehyde. Fixed cells were permeabilized with 0.1% Triton X-100 or 0.1% saponin, and immunofluorescence labeling was performed according to standard procedures [46]. Polyclonal rabbit and monoclonal mouse anti-*T. whipplei* Abs were generated in our laboratory. Rat and mouse Abs specific for Lamp-1 (clone 1D4B and H4A3) were purchased from DSHB (Iowa, USA). Rabbit Ab specific for cathepsin D was a gift from Dr. S. Kornfeld (Washington University School of Medicine, St. Louis, MO) and Rabbit Ab specific for p62 was a gift from S. Meresse (Centre d'Immunologie de Marseille Luminy, Marseille, France). Secondary Alexa Abs, goat anti-mouse IgG conjugated with Alexa 488, goat anti-rat IgG and anti-rabbit IgG coupled with Alexa 555 were purchased from Invitrogen. In human monocytes and macrophages, the colocalization of *T. whipplei* with Lamp-1 or cathepsin D was studied using the couples mouse Lamp-1 Abs with rabbit anti-*T. whipplei* Abs and rabbit anti-cathepsin D Abs with mouse anti-*T. whipplei* Abs, respectively. In BMDMs, the combination of mouse anti-*T. whipplei* Abs, rat anti-Lamp-1 Abs and rabbit anti-cathepsin D Abs was used to directly assess the colocalization of *T. whipplei* with both Lamp-1 and cathepsin D. Cells were then examined by laser scanning microscopy using a confocal microscope (Leica TCS SP5, Heidelberg, Germany) with a 63X/1.32-0.6 oil objective and an electronic Zoom 1.5X. Optical sections of fluorescent images were collected at 0.15-µm intervals using Leica Confocal Software and processed using Adobe Photoshop© V7.0.1 software. At least 100 cells were examined for each experimental condition. The results are expressed as the percentage of bacteria that colocalized with fluorescent markers. Cells were selected as follows: 25 microscope fields, with at least four cells per field containing at least three phagosomes were randomly selected. More than 300 phagosomes were examined per experimental condition.

### Microarray analysis

BMDMs were infected with *T. whipplei* (50 bacteria/cell) for six hours, and total RNA was extracted using the RNeasy Mini Kit (Qiagen). The quality and the quantity of prepared RNA were assessed using the 2100 Bioanalyzer (Agilent Technologies). Sample labeling and hybridization were performed according to the manufacturer's recommendations (One-Color Microarray-Based Gene Expression Analysis). Briefly, labeled cDNA was synthesized using the Low RNA Input Fluorescent Amplification Kit (Agilent Technologies) with 300 ng of total RNA and cyanine 3-labeled CTP. Hybridizations of 4X44k Mouse Whole Genome microarrays (Agilent Technologies) were performed in triplicate for 17 hours at 65°C using the *In situ* Hybridization Kit Plus (Agilent Technologies). Slides were scanned at a 5-µm resolution by a G2505B DNA microarray scanner (Agilent Technologies), and images were analyzed with the Agilent Feature Extractor Software 9.5.1.1. Global normalization by trimmed means was applied to raw datasets using the Excel software (Microsoft). A threshold-free functional profiling of significant features of the microarray was applied to avoid any loss of information as previously described [47]. Significant features were selected by applying the Student's *t* test to the input data with a *p* value<0.01. GO annotation and enrichment of GO biological process were performed with the freely available online tools FatiGO Search and Fatiscan (http://babelomics.bioinfo.cipf.es/). The two-tailed Fisher's exact test was used to determine functional classes of genes significantly over-represented or under-represented (*p*<0.05).

### GEO Database

All data are MIAME compliant and the raw data have been deposited in a MIAME compliant database (GEO). All transcriptional profile files are available in the GEO database at NCBI (accession number GSE20210).

### Immunoassays

Macrophages were treated with or without rhIFNγ for 16 hours and then infected with *T. whipplei* (50 bacteria/cell) for 48 hours in the presence or absence of IFNγ. Cell supernatants were assessed for the presence of IL-16 by immunoassay (R&D Systems). The sensitivity of kits is about 6.2 pg/ml.

### Statistical analysis

Results are expressed as means ± SEM and were analyzed by the non-parametric Mann-Whitney *U* test. Differences were considered significant when *p*<0.05.

## Supporting Information

Figure S1 Effect of IL-16 on the intracellular localization of latex beads. Macrophages were pretreated with rhIL-16 for 18 hours, incubated with latex beads (dilution 1/5000, Sigma Aldrich) for 4 hours, washed to remove unphagocytosed beads and incubated for additional time periods. The intracellular localization of the latex beads was analyzed by indirect immunofluorescence and laser scanning microscopy. The percentage of beads that colocalized with (A) Lamp-1 or (B) cathepsin D was determined. More than 300 phagosomes were examined per experimental condition, and the results are expressed as the mean ± SEM of two independent experiments.
(0.56 MB TIF)

Figure S2 Analysis of transcriptional responses of BMDMs to LPS. BMDMs were stimulated with LPS (100 ng/ml) for six hours, and host responses were analyzed by microarrays. Gene expression values were normalized by trimmed means. Significant features were then compared between wt (blue) and IL-16−/− (green) BMDMs and represented by a Venn diagram. Common significant features are displayed in grey.
(0.58 MB TIF)

Figure S3 Effect of IL-10 on *T. whipplei* replication. Monocytes and macrophages were pretreated with or without rhIL-10 (10 ng/ml) for 18 hours, incubated with *T. whipplei* (50 bacteria/cell) for 4 hours, washed to remove unphagocytosed bacteria and incubated for additional time periods. *T. whipplei* uptake (inset) and replication in monocytes (A) and macrophages (B) were assessed by determining the bacterial DNA copy number by qPCR. The results are expressed as the mean ± SEM of four independent experiments.
(0.53 MB TIF)

Figure S4 Effect of IL-16 on *C. burnetii* replication and intracellular localization. Human macrophages were pretreated with or without rhIL-16 (10 ng/ml) for 18 hours, incubated with *C. burnetii* (200 bacteria/cell) for 4 hours, washed to remove unphagocytosed bacteria and incubated for additional time periods. (A) *C. burnetii* replication was assessed by determining the bacterial DNA copy number by qPCR. The results are expressed as the mean ± SEM of three independent experiments. (B) The intracellular localization of *C. burnetii* within IL-16 treated cells was assessed by indirect immunofluorescence and laser scanning microscopy. The percentage of organisms that colocalized with lysosomes (Lamp-1 and cathepsin D) was determined. More than 150 phagosomes were examined per experimental condition, and the results are expressed as the mean ± SEM of three independent experiments.
(0.52 MB TIF)

Figure S5 Effect of IL-10 on T. whipplei intracellular localization. Monocytes (A, B) and macrophages (C, D) were pretreated with or without IL-10 (10 ng/ml) for 18 hours, incubated with T. whipplei for 4 hours (50 bacteria/cell), washed to remove unphagocytosed bacteria and incubated for additional time periods. The intracellular localization of *T. whipplei* within IL-10-treated cells was assessed by indirect immunofluorescence and laser scanning microscopy. The percentage of organisms that colocalized with Lamp-1 (A and C) or cathepsin D (B and D) was determined. More than 300 phagosomes were examined per experimental condition, and the results are expressed as the mean ± SEM of five independent experiments.
(0.76 MB TIF)

Figure S6 IL-16 does not modulate molecules involved in phagosome conversion. Macrophages treated with rhIL-16 (10 ng/ml) for different time periods were lysed and RNA was extracted using the QIAamp RNA Mini Kit (Qiagen). cDNA was synthesized from 1 µg of total RNA using SuperScript II RNase H reverse transcriptase (Invitrogen). Specific primers for each gene were designed using the Primer3Plus software (http://frodo.wi.mit.edu/primer3/). The primer sequences were as follows: for Rab5, cgggccaaatactggaaata (left primer) and aggacttgcttgcctctgaa (right primer); for Rab7, ggccttctacagaggtgcag (left primer) and ccggtcattcttgtccagtt (right primer); for β-actin used as an internal control, ggaaatcgtgcgtgacatta (left primer) and aggaaggaaggctggaagag (right primer). PCR was performed using Hotstart Taq polymerase (Qiagen) following the manufacturer's recommendations. PCR products were electrophoresed through a 1% agarose gel containing ethidium bromide. Data were acquired with a Gel Doc 2000 (BioRad), and gene expression was normalized to β-actin. The figure is representative of three experiments. (B) Macrophages were stimulated with or without rhIL-16 (10 ng/ml) for 16 hours and washed with ice-cold PBS. Western blotting was performed as previously described (Al Moussawi et al. 2010). In brief, cells were lysed in ice-cold RIPA buffer containing protease inhibitor (Complete, Roche) and phosphatase inhibitor (Phosphostop, Roche) cocktails. After clearing, cell lysates were loaded onto 12% SDS polyacrylamide gels, electrophoresed and transferred onto nitrocellulose membranes (Millipore). The membranes were incubated with primary Abs directed against α-tubulin (Cell Signaling), Lamp-1 (H4A3, Abcam) or cathepsin D and then incubated with peroxidase-conjugated Abs directed against anti-rabbit or anti-mouse IgG (Pierce). The blots were then revealed using the Immobilon Western Chemiluminescent HRP substrate (Millipore). Each blot is a representative of three independent experiments.
(0.68 MB TIF)

Table S1 Transcripts significantly induced by *T. whipplei* in both wt and IL-16−/− BMDMs.
(0.08 MB DOC)

Table S2 IFNγ induces *T. whipplei* elimination in a dose-dependent manner. Macrophages were treated with different concentrations of rhIFNγ and infected with *T. whipplei* (50 bacteria/cell). Bacterial replication was assessed by determining the bacterial DNA copy number by qPCR and cell viability was determined using Trypan blue exclusion. The results are expressed as the mean ± SEM of four independent experiments performed in triplicate.
(0.03 MB DOC)
