# Supplementary figures and images for: IL-16 Promotes T. whipplei Replication by Inhibiting Phagosome Conversion and Modulating Macrophage Activation
Source: PLoS One. 2010 Oct 21;5(10):e13561. doi: 10.1371/journal.pone.0013561 (PMC2958842; doi:10.1371/journal.pone.0013561)

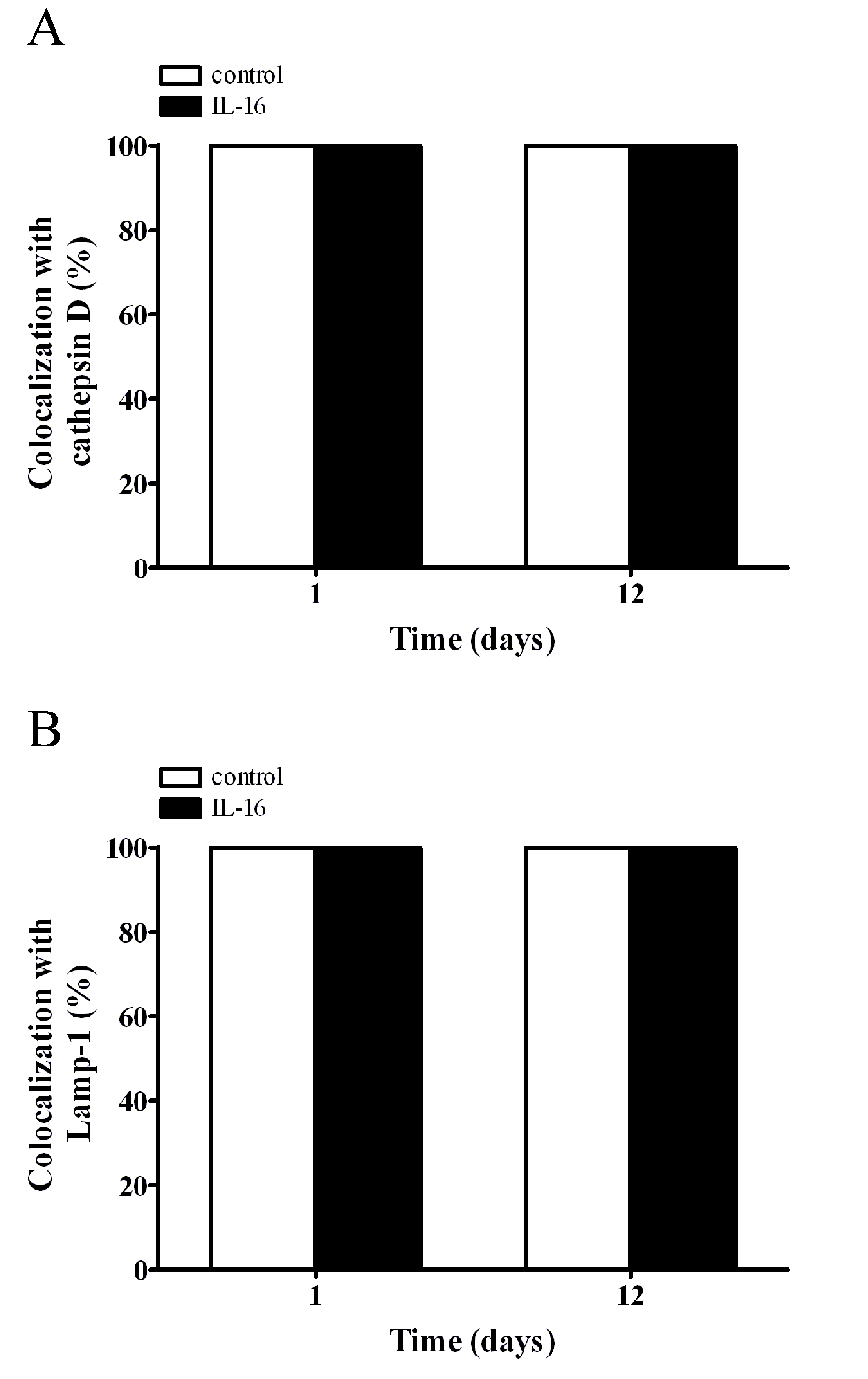

Supplement: Figure S1 — Effect of IL-16 on the intracellular localization of latex beads. Macrophages were pretreated with rhIL-16 for 18 hours, incubated with latex beads (dilution 1/5000, Sigma Aldrich) for 4 hours, washed to remove unphagocytosed beads and incubated for additional time periods. The intracellular localization of the latex beads was analyzed by indirect immunofluorescence and laser scanning microscopy. The percentage of beads that colocalized with (A) Lamp-1 or (B) cathepsin D was determined. More than 300 phagosomes were examined per experimental condition, and the results are expressed as the mean ± SEM of two independent experiments. (0.56 MB TIF) [file pone.0013561.s001.tif]

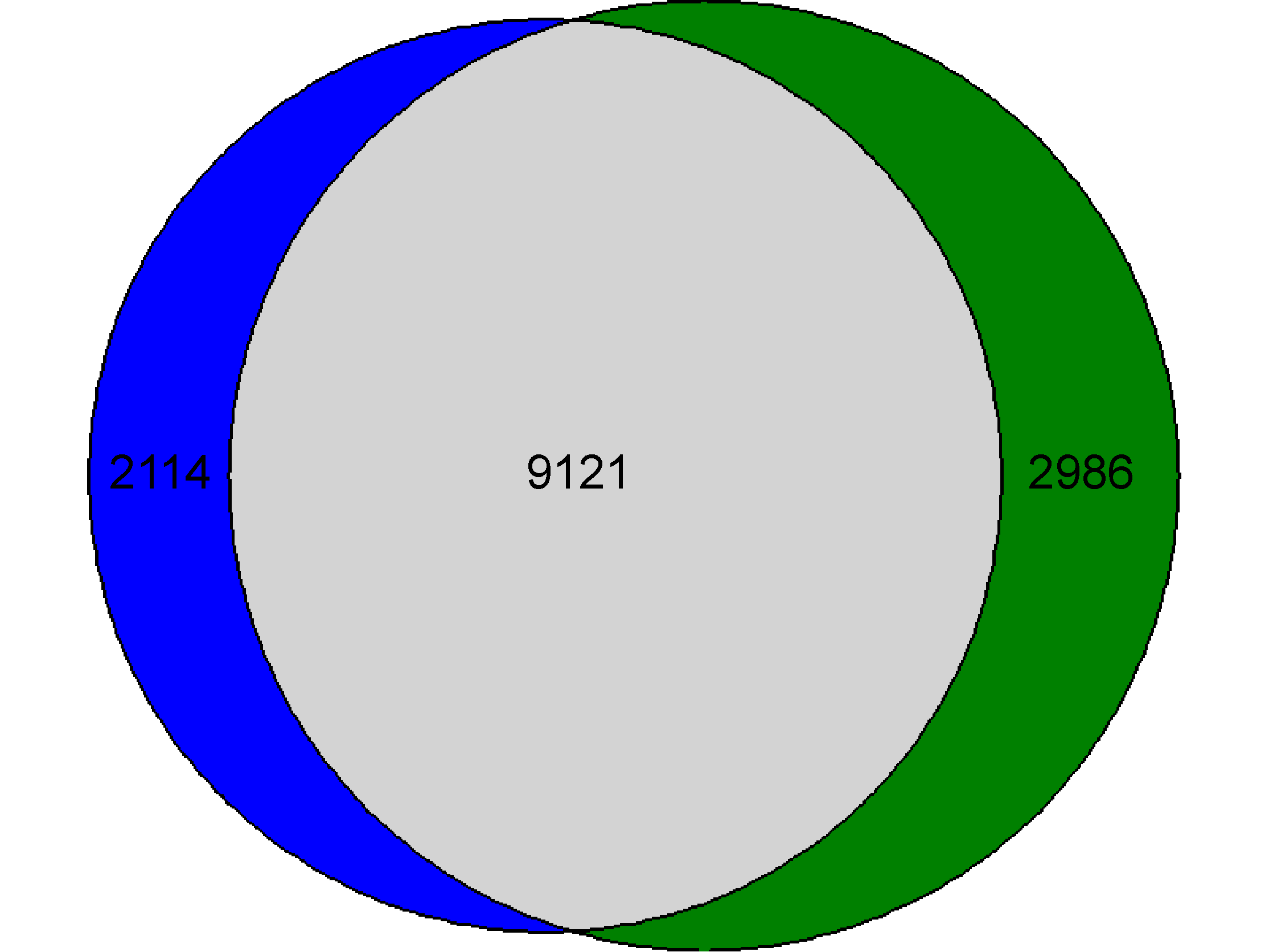

Supplement: Figure S2 — Analysis of transcriptional responses of BMDMs to LPS. BMDMs were stimulated with LPS (100 ng/ml) for six hours, and host responses were analyzed by microarrays. Gene expression values were normalized by trimmed means. Significant features were then compared between wt (blue) and IL-16−/− (green) BMDMs and represented by a Venn diagram. Common significant features are displayed in grey. (0.58 MB TIF) [file pone.0013561.s002.tif]

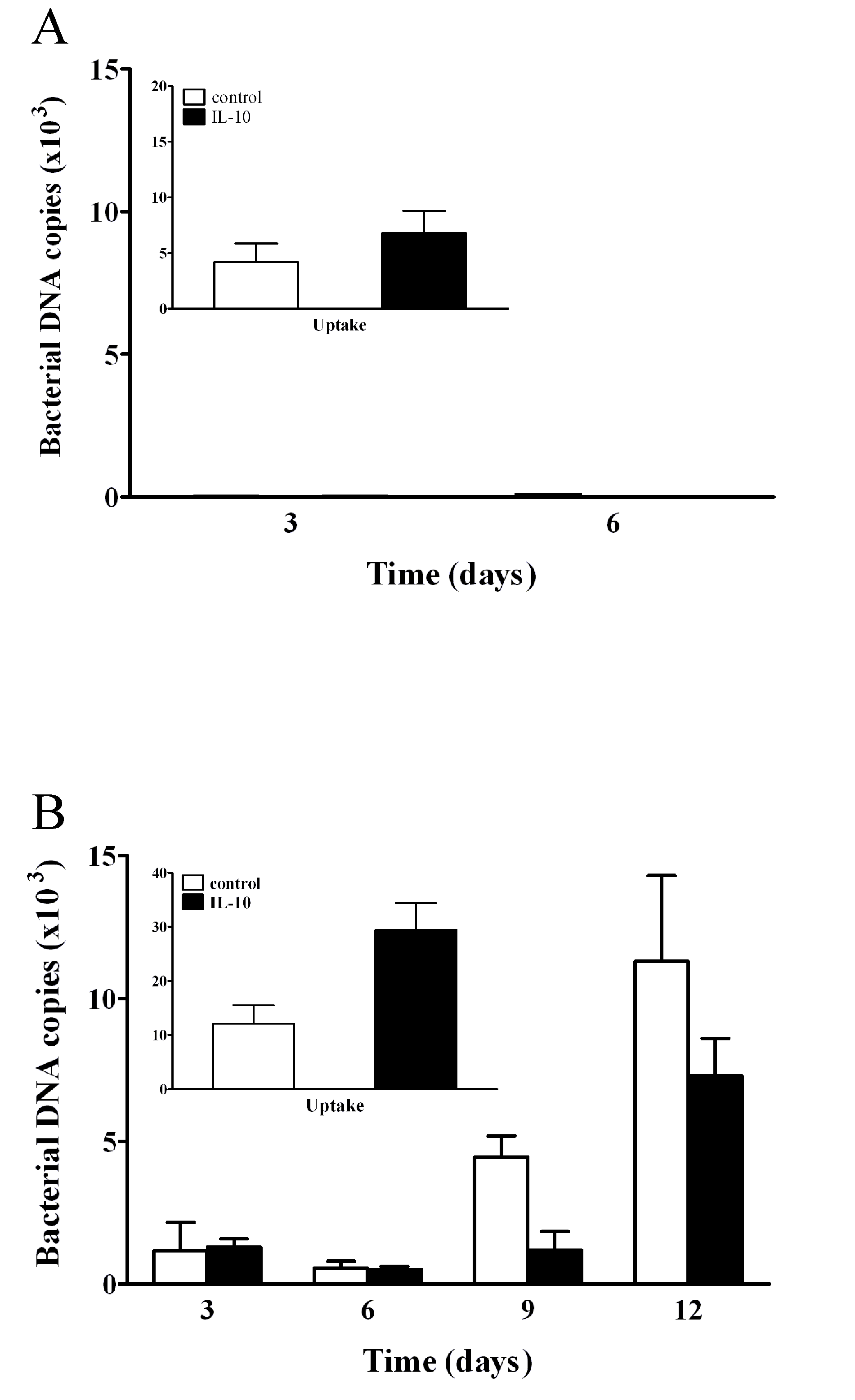

Supplement: Figure S3 — Effect of IL-10 on T. whipplei replication. Monocytes and macrophages were pretreated with or without rhIL-10 (10 ng/ml) for 18 hours, incubated with T. whipplei (50 bacteria/cell) for 4 hours, washed to remove unphagocytosed bacteria and incubated for additional time periods. T. whipplei uptake (inset) and replication in monocytes (A) and macrophages (B) were assessed by determining the bacterial DNA copy number by qPCR. The results are expressed as the mean ± SEM of four independent experiments. (0.53 MB TIF) [file pone.0013561.s003.tif]

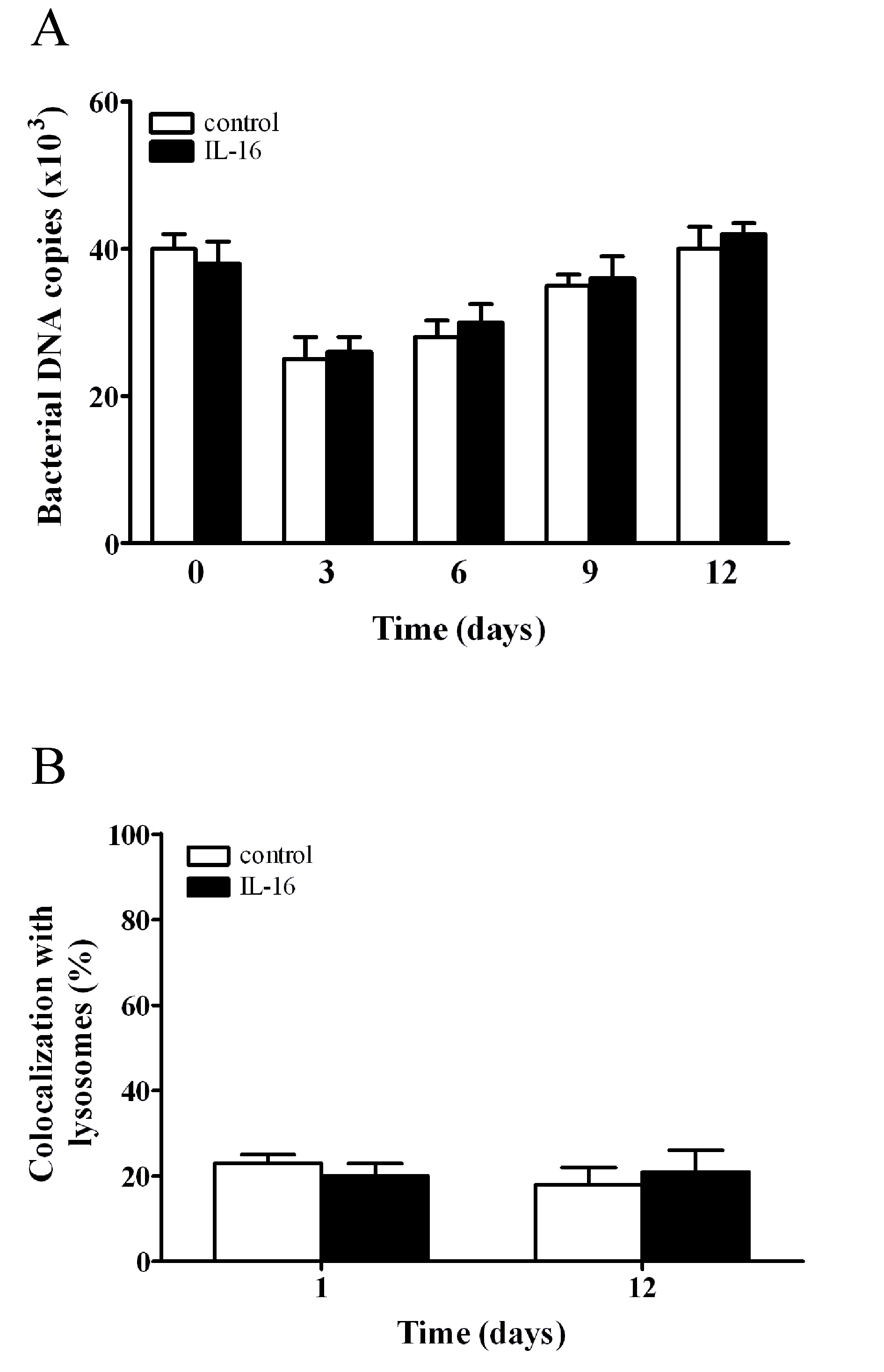

Supplement: Figure S4 — Effect of IL-16 on C. burnetii replication and intracellular localization. Human macrophages were pretreated with or without rhIL-16 (10 ng/ml) for 18 hours, incubated with C. burnetii (200 bacteria/cell) for 4 hours, washed to remove unphagocytosed bacteria and incubated for additional time periods. (A) C. burnetii replication was assessed by determining the bacterial DNA copy number by qPCR. The results are expressed as the mean ± SEM of three independent experiments. (B) The intracellular localization of C. burnetii within IL-16 treated cells was assessed by indirect immunofluorescence and laser scanning microscopy. The percentage of organisms that colocalized with lysosomes (Lamp-1 and cathepsin D) was determined. More than 150 phagosomes were examined per experimental condition, and the results are expressed as the mean ± SEM of three independent experiments. (0.52 MB TIF) [file pone.0013561.s004.tif]

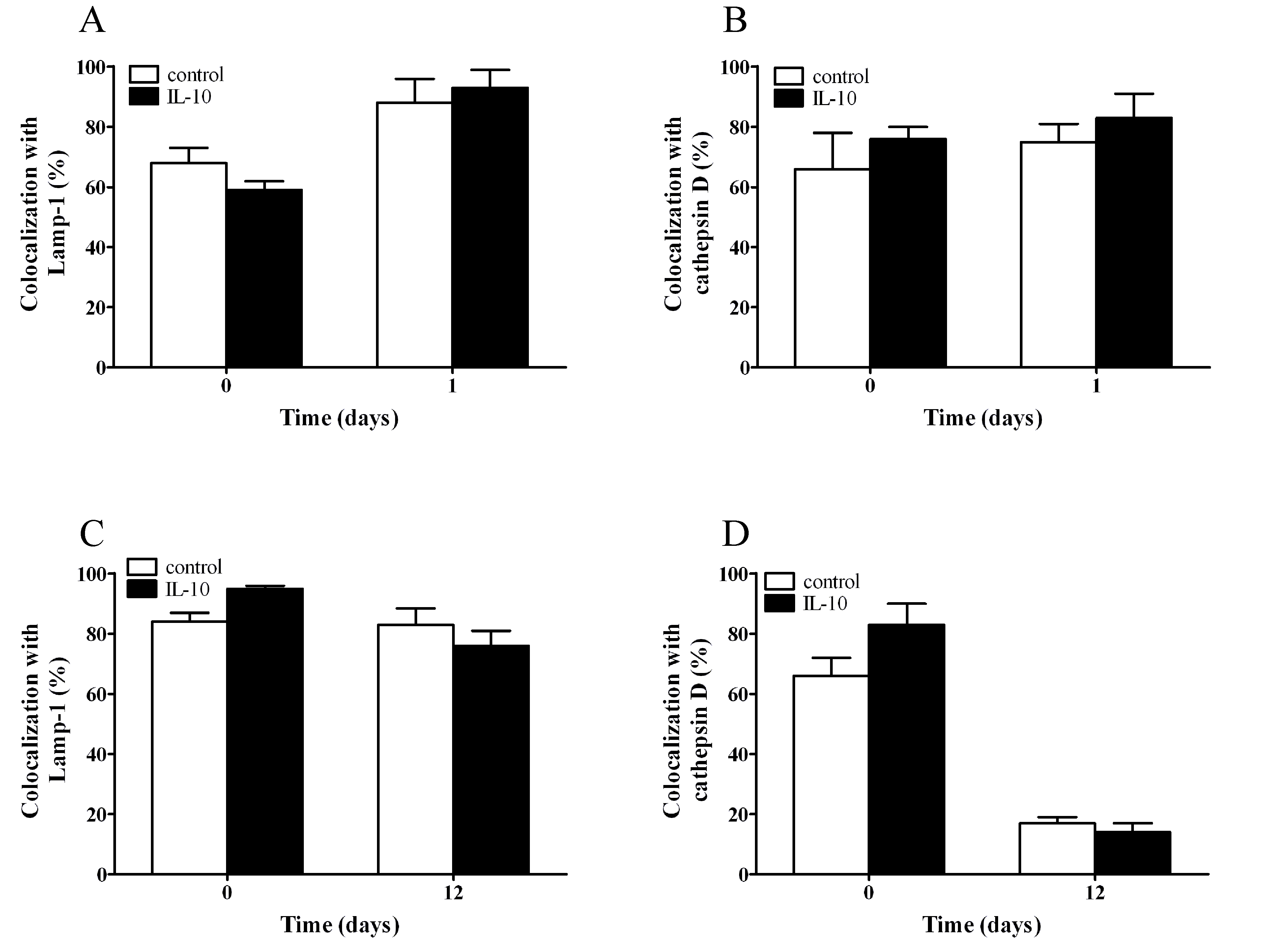

Supplement: Figure S5 — Effect of IL-10 on T. whipplei intracellular localization. Monocytes (A, B) and macrophages (C, D) were pretreated with or without IL-10 (10 ng/ml) for 18 hours, incubated with T. whipplei for 4 hours (50 bacteria/cell), washed to remove unphagocytosed bacteria and incubated for additional time periods. The intracellular localization of T. whipplei within IL-10-treated cells was assessed by indirect immunofluorescence and laser scanning microscopy. The percentage of organisms that colocalized with Lamp-1 (A and C) or cathepsin D (B and D) was determined. More than 300 phagosomes were examined per experimental condition, and the results are expressed as the mean ± SEM of five independent experiments. (0.76 MB TIF) [file pone.0013561.s005.tif]

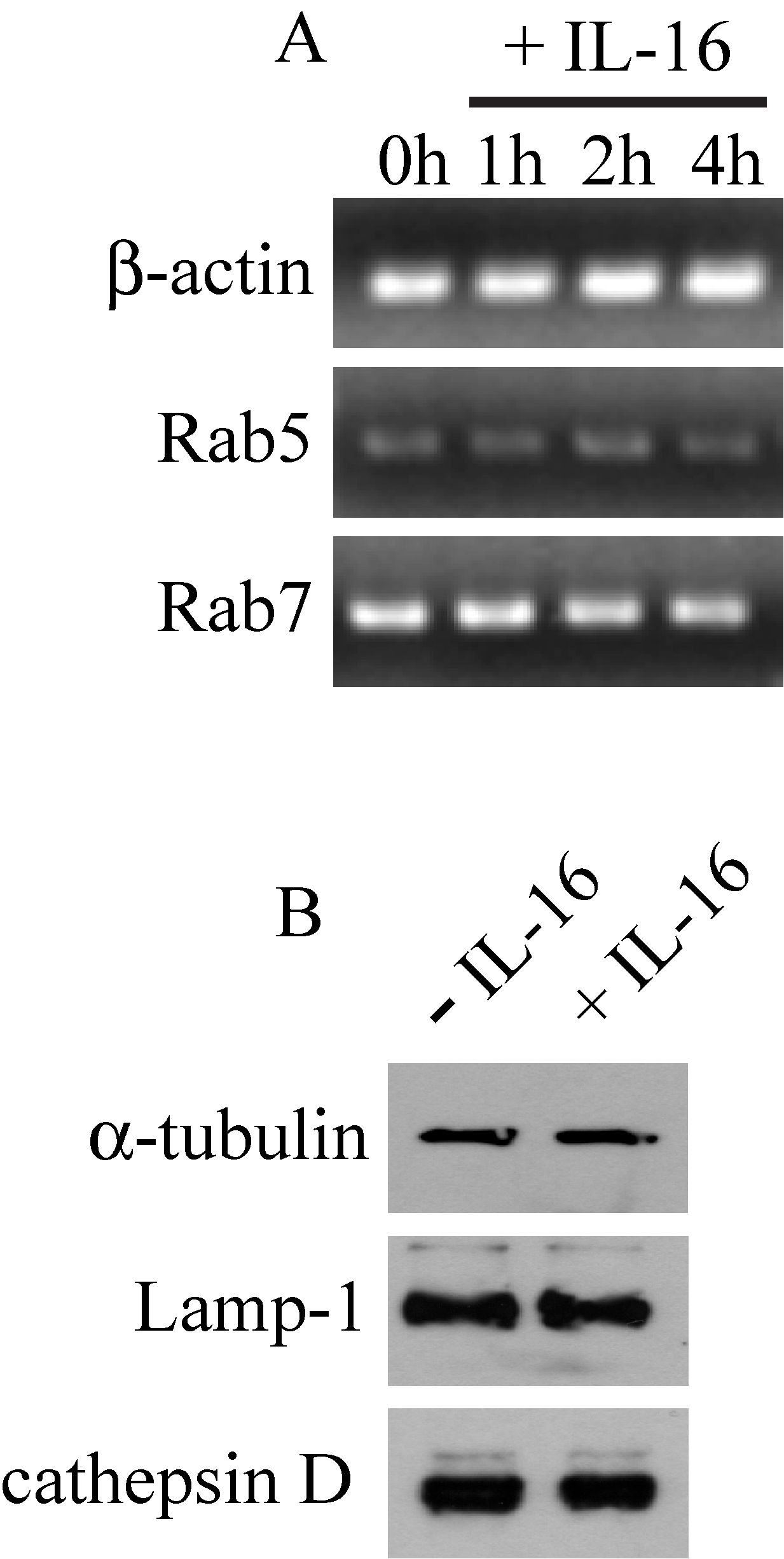

Supplement: Figure S6 — IL-16 does not modulate molecules involved in phagosome conversion. Macrophages treated with rhIL-16 (10 ng/ml) for different time periods were lysed and RNA was extracted using the QIAamp RNA Mini Kit (Qiagen). cDNA was synthesized from 1 µg of total RNA using SuperScript II RNase H reverse transcriptase (Invitrogen). Specific primers for each gene were designed using the Primer3Plus software (http://frodo.wi.mit.edu/primer3/). The primer sequences were as follows: for Rab5, cgggccaaatactggaaata (left primer) and aggacttgcttgcctctgaa (right primer); for Rab7, ggccttctacagaggtgcag (left primer) and ccggtcattcttgtccagtt (right primer); for β-actin used as an internal control, ggaaatcgtgcgtgacatta (left primer) and aggaaggaaggctggaagag (right primer). PCR was performed using Hotstart Taq polymerase (Qiagen) following the manufacturer's recommendations. PCR products were electrophoresed through a 1% agarose gel containing ethidium bromide. Data were acquired with a Gel Doc 2000 (BioRad), and gene expression was normalized to β-actin. The figure is representative of three experiments. (B) Macrophages were stimulated with or without rhIL-16 (10 ng/ml) for 16 hours and washed with ice-cold PBS. Western blotting was performed as previously described (Al Moussawi et al. 2010). In brief, cells were lysed in ice-cold RIPA buffer containing protease inhibitor (Complete, Roche) and phosphatase inhibitor (Phosphostop, Roche) cocktails. After clearing, cell lysates were loaded onto 12% SDS polyacrylamide gels, electrophoresed and transferred onto nitrocellulose membranes (Millipore). The membranes were incubated with primary Abs directed against α-tubulin (Cell Signaling), Lamp-1 (H4A3, Abcam) or cathepsin D and then incubated with peroxidase-conjugated Abs directed against anti-rabbit or anti-mouse IgG (Pierce). The blots were then revealed using the Immobilon Western Chemiluminescent HRP substrate (Millipore). Each blot is a representative of three independent experiments. (0.6 [file pone.0013561.s006.tif]
