# Supplementary material for: IL-16 Promotes T. whipplei Replication by Inhibiting Phagosome Conversion and Modulating Macrophage Activation
Source: PLoS One. 2010 Oct 21;5(10):e13561. doi: 10.1371/journal.pone.0013561 (PMC2958842; doi:10.1371/journal.pone.0013561)
Supplement: Table S1 — Transcripts significantly induced by T. whipplei in both wt and IL-16−/− BMDMs. (0.08 MB DOC) [file pone.0013561.s007.doc]

**Table S1.** Transcripts significantly induced by *T. whipplei* in both wt and IL-16^-/-^ BMDMs

| **Gene name** | **Systematic name** | **Log2 FC (wt)** | ***p* value (wt)** | **Log2 FC (IL-16^-/-^)** | ***p* value (IL-16^-/-^)** |
| --- | --- | --- | --- | --- | --- |
| Irg1 | AK152177 | 2.75 | 1.94 x 10^-4^ | 1.39 | 3.93 x 10^-3^ |
| Olr1 | NM_138648 | 2.75 | 2.45 x 10^-3^ | 2.70 | 4.17 x 10^-4^ |
| Irg1 | L38281 | 2.55 | 6.90 x 10^-5^ | 1.35 | 6.26 x 10^-3^ |
| Mmp14 | NM_008608 | 1.62 | 7.18 x 10^-4^ | 1.42 | 5.33 x 10^-4^ |
| C79127 | NM_177691 | 1.55 | 8.07 x 10^-3^ | 1.33 | 9.96 x 10^-3^ |
| Clec4e | NM_019948 | 1.36 | 1.87 x 10^-3^ | 1.15 | 3.32 x 10^-6^ |
| Ccl17 | NM_011332 | 1.21 | 2.62 x 10^-3^ | 1.41 | 3.79 x 10^-3^ |
| Ccrl2 | NM_017466 | 1.20 | 3.84 x 10^-3^ | 1.05 | 1.10 x 10^-4^ |
| Traf1 | NM_009421 | 1.13 | 3.88 x 10^-3^ | 0.49 | 4.30 x 10^-4^ |
| Spic | NM_011461 | 1.13 | 1.04 x 10^-3^ | 0.82 | 6.05 x 10^-3^ |
| Gpr84 | NM_030720 | 1.12 | 1.03 x 10^-3^ | 0.74 | 1.88 x 10^-3^ |
| Icam1 | BC008626 | 1.05 | 1.30 x 10^-3^ | 1.08 | 1.46 x 10^-3^ |
| Tlr2 | NM_011905 | 1.04 | 2.54 x 10^-3^ | 0.92 | 6.36 x 10^-3^ |
| Nfkbie | NM_008690 | 1.02 | 2.63 x 10^-4^ | 0.92 | 5.60 x 10^-3^ |
| Src | NM_009271 | 0.96 | 3.73 x 10^-4^ | 0.84 | 8.49 x 10^-3^ |
| 4933430I17Rik | NM_177607 | 0.96 | 3.11 x 10^-3^ | 0.65 | 4.96 x 10^-4^ |
| Mefv | NM_019453 | 0.96 | 5.97 x 10^-3^ | 1.03 | 4.61 x 10^-3^ |
| F10 | NM_007972 | 0.89 | 2.79 x 10^-4^ | 0.81 | 2.29 x 10^-3^ |
| Ralgds | NM_009058 | 0.82 | 2.85 x 10^-3^ | 0.85 | 4.73 x 10^-3^ |
| Slc2a6 | NM_172659 | 0.73 | 9.51 x 10^-3^ | 0.78 | 4.64 x 10^-3^ |
| C3 | NM_009778 | 0.73 | 6.58 x 10^-3^ | 0.63 | 7.03 x 10^-3^ |
| Tnf | NM_013693 | 0.72 | 5.60 x 10^-3^ | 0.21 | 1.03 x 10^-3^ |
| 5730528L13Rik | NM_028137 | 0.68 | 2.72 x 10^-3^ | 0.47 | 6.57 x 10^-3^ |
| Nfkbia | NM_010907 | 0.65 | 9.93 x 10^-3^ | 0.31 | 7.41 x 10^-4^ |
| Mcoln2 | NM_026656 | 0.58 | 9.07 x 10^-3^ | 0.59 | 4.50 x 10^-4^ |
| Ccl24 | NM_019577 | 0.58 | 4.63 x 10^-3^ | 0.40 | 4.36 x 10^-3^ |
| Tnfrsf1b | NM_011610 | 0.49 | 8.52 x 10^-3^ | 0.56 | 5.67 x 10^-3^ |
| Zmynd15 | NM_001029929 | 0.47 | 3.50 x 10^-3^ | 0.58 | 7.18 x 10^-3^ |
| Cdc42ep2 | NM_026772 | 0.40 | 6.31 x 10^-3^ | 0.62 | 8.60 x 10^-3^ |
| Bcl3 | NM_033601 | 0.37 | 7.58 x 10^-3^ | 0.32 | 3.60 x 10^-3^ |
| Abcc5 | NM_013790 | 0.36 | 8.25 x 10^-3^ | 0.12 | 2.09 x 10^-5^ |
| Znrf1 | NM_133206 | 0.31 | 2.75 x 10^-3^ | 0.30 | 1.64 x 10^-3^ |
| Gcdh | NM_008097 | -0.20 | 5.22 x 10^-3^ | -0.20 | 2.76 x 10^-3^ |
| Fbxo5 | NM_025995 | -0.40 | 5.34 x 10^-3^ | -0.41 | 4.37 x 10^-3^ |
| Rttn | NM_175542 | -0.42 | 2.36 x 10^-3^ | -0.59 | 2.20 x 10^-3^ |
| Cdca5 | NM_026410 | -0.48 | 4.33 x 10^-3^ | -0.33 | 6.27 x 10^-3^ |
| Il1rl1 | NM_001025602 | -0.66 | 1.70 x 10^-3^ | -0.58 | 6.81 x 10^-3^ |
| Tcf19 | NM_025674 | -0.67 | 2.56 x 10^-3^ | -0.44 | 3.11 x 10^-3^ |
| BC031353 | NM_153584 | -0.72 | 5.40 x 10^-3^ | -0.67 | 9.77 x 10^-3^ |
| 4930430E16Rik | NM_028672 | -0.76 | 9.98 x 10^-3^ | -0.91 | 1.11 x 10^-4^ |
| Map2k6 | NM_011943 | -0.83 | 1.03 x 10^-3^ | -0.60 | 7.43 x 10^-4^ |
| Rasgrp3 | NM_207246 | -0.83 | 2.41 x 10^-3^ | -0.66 | 4.28 x 10^-3^ |
