# Supplementary material for: IL-16 Promotes T. whipplei Replication by Inhibiting Phagosome Conversion and Modulating Macrophage Activation
Source: PLoS One. 2010 Oct 21;5(10):e13561. doi: 10.1371/journal.pone.0013561 (PMC2958842; doi:10.1371/journal.pone.0013561)
Supplement: Table S2 — IFNγ induces T. whipplei elimination in a dose-dependent manner. Macrophages were treated with different concentrations of rhIFNγ and infected with T. whipplei (50 bacteria/cell). Bacterial replication was assessed by determining the bacterial DNA copy number by qPCR and cell viability was determined using Trypan blue exclusion. The results are expressed as the mean ± SEM of four independent experiments performed in triplicate. (0.03 MB DOC) [file pone.0013561.s008.doc]

**Table S2. IFNγ induces *T. whipplei* elimination in a dose-dependent manner**

|  | Bacterial DNA copies (x10^3^) | | Cell viability (%) |
| --- | --- | --- | --- |
| IFNγ (UI/ml) | day 0 | day 12 | day 12 |
| 0 | 12.1 ± 3.4 | 11.3 ± 3 | 96 ± 4 |
| 250 | 15 ± 1.8 | 5.2 ± 2.8 | 92 ± 6 |
| 500 | 22.6 ± 6 | 1.7 ± 0.26 | 95 ± 2 |
| 1000 | 21 ± 5.2 | 0 | 66 ± 13 |
